# Profile and triage validity of trauma patients triaged green: a prospective cohort study from a secondary care hospital in India

**DOI:** 10.1136/bmjopen-2022-065036

**Published:** 2023-05-08

**Authors:** Aroke Anna Anthony, Rohini Dutta, Bhakti Sarang, Siddarth David, Gerard O'Reilly, Nakul P Raykar, Monty Khajanchi, Jonatan Attergrim, Kapil Dev Soni, Naveen Sharma, Monali Mohan, Anita Gadgil, Nobhojit Roy, Martin Gerdin Wärnberg

**Affiliations:** 1 Doctors For You, Mumbai, India; 2 World Health Organization Collaborating Center for Research in Surgical Care Delivery in Low-and-Middle Income Countries, Mumbai, India; 3 Department of Global Health and Social Medicine, Harvard Medical School, Boston, Massachusetts, USA; 4 Department of Surgery, Terna Medical College & Hospital, New Mumbai, India; 5 Department of Global Public Health, Karolinska Institutet, Solna, Sweden; 6 Department of Emergency Medicine, Monash University, Clayton, Victoria, Australia; 7 Department of Emergency Surgery, Brigham and Women’s Hospital, Boston, Massachusetts, USA; 8 Department of Surgery, Seth Gowardhandas Sunderdas Medical College and King Edward Memorial Hospital, Mumbai, India; 9 Function Perioperative Medicine and Intensive Care, Karolinska University Hospital, Solna, Sweden; 10 Critical Care, All India Institute of Medical Sciences, New Delhi, India; 11 Department of Surgery, All India Institute of Medical Sciences, Jodhpur, Rajasthan, India

**Keywords:** trauma management, accident & emergency medicine, trauma management

## Abstract

**Objectives:**

To evaluate the profile of non-urgent patients triaged ‘green’, as part of a triage trial in the emergency department (ED) of a secondary care hospital in India. The secondary aim was to validate the triage trial with the South African Triage Score (SATS).

**Design:**

Prospective cohort study.

**Setting:**

A secondary care hospital in Mumbai, India.

**Participants:**

Patients aged 18 years and above with a history of trauma defined as having any of the external causes of morbidity and mortality listed in block V01–Y36, chapter XX of the International Classification of Disease version 10 codebook, triaged green between July 2016 and November 2019.

**Primary and secondary outcome measures:**

Outcome measures were mortality within 24 hours, 30 days and mistriage.

**Results:**

We included 4135 trauma patients triaged green. The mean age of patients was 32.8 (±13.1) years, and 77% were males. The median (IQR) length of stay of admitted patients was 3 (13) days. Half the patients had a mild Injury Severity Score (3–8), with the majority of injuries being blunt (98%). Of the patients triaged green by clinicians, three-quarters (74%) were undertriaged on validating with SATS. On telephonic follow-up, two patients were reported dead whereas one died while admitted in hospital.

**Conclusions:**

Our study highlights the need for implementation and evaluation of training in trauma triage systems that use physiological parameters, including pulse, systolic blood pressure and Glasgow Coma Scale, for the in-hospital first responders in the EDs.

**STRENGTHS AND LIMITATIONS OF THIS STUDY**

- This is a prospective cohort study, with vital signs recorded by a dedicated research officer, documenting the profile of green triaged patients from a public secondary care hospital in an urban LMIC setting conducted over a period of 3 years.
- Triage validity was assessed using a standardised and validated triage scoring system (South African Triage Score) that included both physiological parameters and injury characteristics of the patients.
- The study provides data from a single secondary care hospital in Mumbai. Therefore, the results cannot be generalised to other Indian hospitals due to hospital bias.
- Only the first 10 consecutive patients’ data were collected each shift during the study period.
- Data on 30-day mortality were missing for some patients while we have no data on patient morbidity.

## Introduction

Globally, 4.4 million people die from trauma annually, with India contributing to 20% of this burden.^1^ Trauma represents the second most common cause of death after age 5 in India with the majority of deaths attributable to road traffic-related deaths.^2 3^ With 50% of deaths due to trauma occurring in the hospitals, there in an urgent need to strengthen in-hospital care for trauma patients.^4^

Trauma care is highly time-sensitive.^5^ Hospital triage systems can ensure that critically ill patients are identified and receive care promptly.^6^ Several triage scores are used across different countries and hospital settings.^7 8^

India’s high population density, poorly developed prehospital care and a lack of appropriate referral systems leads to overcrowding in the emergency departments (EDs).^9–12^ Most EDs lack triage protocols and the level of emergency patient care is decided by clinicians who are not trained specifically in trauma care.^13 14^ The overcrowding diverts resources from patients requiring immediate care.

In our study, clinicians at a triage-naive ED were introduced to a triage trial, as part of a multicentre triage project, the Trauma Triage Study in India (TTRIS). TTRIS compared prediction models for triage in adult trauma patients presenting to various EDs across India.^15^ In TTRIS, the patients were tacitly designated one of the four trauma triage categories by clinicians, based on their understanding of trauma triage; into red, orange, yellow, green, with red and green denoting the most and least urgent patient status, respectively.

We aimed to evaluate the profile of the non-urgent patients who were triaged green by clinicians and retrospectively compare the validity of this category using the South African Triage Score (SATS).^16 17^

## Methodology

### Study design

This single-centre prospective cohort study between July 2016 and November 2019 is part of the TTRIS which compares prediction models for triage in adult trauma patients presenting to various EDs across India.

### Study setting

The study site was the ED of Khurshedji Behramji Bhabha Hospital (KBBH), a 436 bedded regional secondary healthcare centre located in Mumbai, India, catering to approximately 350 patients each day in the ED. It is a public hospital with free or nominal fees, providing access to low socioeconomic groups and receives patients from across the city. At KBBH, trauma care is imparted as a subspecialty along with medical, surgical and obstetric care. The hospital has an intensive care facility but there is no neurosurgery department, so patients in need of neurosurgical management are referred to tertiary care centres. Plain radiography and ultrasonography are available round the clock; however, CT is only available in-house from 7:00 to 18:00 hour. The patients arriving at the ED are first seen by a casualty medical officer largely on a first-come, first-served basis without a formalised system of triaging patients.

### Clinician’s tacit triage

As part of data collection of TTRIS, the triage-naive clinicians were only given standard comparable labels to different trauma triage colour categories, without provision to any formal tool or training about the same. The clinicians involved have a minimum of 2 years clinical experience, however, they are neither trained in trauma care as a specialty nor are they necessarily trained in trauma management courses such as Advanced Trauma Life Support. After their initial on-arrival assessment of each patient, the research officers asked the clinicians to categorise the urgency of patients into the aforementioned colour-coded triage groups,^15^ henceforth referred to as the clinician’s tacit triage (CTT). The CTT was just based on the clinician’s experiential and intuitive clinical knowledge. For doing this, the clinicians were allowed to use all available information that was extracted by them during initial routine assessment. The CTTs were not used to determine treatment decisions in the ED as there was no formalised tool or protocol in place for assigning the triage and coupling it with patient management. The clinicians were individually informed about the aim and methodology used for the TTRIS study; however, the clinicians were neither involved in the conception nor were they part of the research team analysing the results.

### Participants

#### Inclusion criteria

We included all the patients aged 18 years and above presenting to the KBBH ED with a history of trauma as their primary complaint and triaged green by CTT on initial evaluation irrespective of their injury severity. A history of trauma was defined here as having any of the external causes of morbidity and mortality listed in block V01–Y36, chapter XX of the International Classification of Disease version 10 (ICD-10) online codebook, with some exclusions (see online supplemental material 1).^18^

10.1136/bmjopen-2022-065036.supp2 Supplementary data

#### Exclusion criteria

Patients with missing data in one or more variables used for analysis or who did not consent to follow-up were excluded from the analysis.

### Source and methods of selection of participants and follow-up

The research officer at KBBH observed morning, evening and night shifts (6-hour observational shifts). These shifts were not aligned with the working hours of the clinical staff to reduce bias and accounting for shift fatigue of the clinicians. Due to the large patient load and time and budgetary constraints of the project, data were collected from the first 10 consecutive patients only, irrespective of their CTT, who presented during each shift. The research officer collected the vital signs but was in no way involved in patient assessment or management.

The research officer performed follow-up at 24 hours, 30 days after arrival at the ED. The follow-up was completed in-person or by telephone, depending on whether the patient was still hospitalised or if the patient had been discharged. The phone numbers of one or more contact persons, mostly relatives, were collected on enrolment and those people were contacted if the participant did not reply to the follow-up telephone calls. The outcome was recorded as missing if neither the patient nor the relative were available for follow-up at the specified time points.

### Variables collected for retrospective assessment

To evaluate the profile of patients triaged green we analysed the 24 hours and 30 days mortality, age, sex, mechanism of injury, injury-related details, assigned CTT level, ward or intensive care unit (ICU) admission status, and physiological measures. The physiological measures were systolic blood pressure (SBP), respiratory rate (RR), heart rate, peripheral capillary oxygen saturation, Glasgow Coma Scale (GCS) and Alert Verbal Pain Unresponsive scale (AVPU).

GCS was categorised into no or mild traumatic brain injury (TBI) (13–15), moderate TBI (9–12), severe TBI (3–8).^19^ Length of stay in the hospital (LoS) was calculated using the data and time of admission in the hospital to the data and time of discharge alive from the hospital, mortality, leave against medical advice (LAMA) or abscond. Injury severity score (ISS) was allocated retrospectively with ‘mild’ (3–8), ‘moderate’ (9–15), ‘severe’ (16–25) and ‘profound’ (>25) categories. Patients for whom ISS could not be coded, for example, when there were no recorded injuries, were assigned ‘no defined ISS’.^20^ The revised trauma score (RTS) which includes GCS, SBP and RR and excludes capillary refill and respiratory expansion, which were difficult to assess in the field was computed and categorised as RTS<4 and RTS>4.^20 21^

Injuries were recorded and coded using ICD-10 in the TTRIS dataset. Patients were divided into categories with respect to the most critical injury namely, crush injury, injury to internal organs, blood vessel injury, amputation, fracture, dislocation, burn, multiple injury, unspecified injury, open wound and superficial injury.^18^ Injury characteristics of patients who presented to the ED with no injuries were categorised as ‘no defined injury’. For patients with multiple injuries, the more critical one was considered for categorising patients as per injury. Time of arrival of patients was categorised into four groups, namely, morning (6:00–11:59 hour), afternoon (12:00–17:59 hour), evening (18:00–23:59 hour) and night (12:00–5:59 hour).^22^

To determine the validity of CTT, we retrospectively used SATS. SATS has two components, Triage Early Warning Score (TEWS) which uses the physiological parameters and the SATS clinical discriminators (SATScd) that use pathology of the patient to triage.^16^ Retrospectively calculated variables and triage categories are henceforth labelled with a prefix r, for example, rSATS. First the rTEWS was calculated and then matched for rSATScd. If a clinical discriminator, such as fracture or dislocation, was present the rSATS was updated to match the triage level assigned to each SATScd (see online supplemental material 2), to be classified into rRed, rOrange, rYellow and rGreen.

10.1136/bmjopen-2022-065036.supp1 Supplementary data

### Bias

There were three layers of quality control. First, data were entered using a dedicated electronic data collection instrument with extensive logical checks and prompts for unlikely but possible values. Second, the collected data were reviewed on a weekly basis and discussed during weekly online conferences with all research officers and the project leads throughout the duration of the data collection period. Third, on-site quality control sessions were conducted every 3–4 months. During these sessions, a second research officer collected data alongside the research officer who worked at the ED. The quality-controlled data were then compared with the standard data.

### Patient and public involvement

No patients were involved in the design of the study.

### Statistical methods

Data were analysed using R statistical software V.4.04.^23^ Complete-case analysis was performed to only include patients with complete data. We describe the sample using frequencies and percentages for categorical variables, and mean and SD for normally distributed continuous variables and median and IQR for non-normally distributed continuous variables. The number of patients triaged green by the SATS was divided by the number of patients triaged green as per clinician triage (4135), the resultant proportion minus one was considered as the proportion of patients mistriaged.

## Results

**Figure 1:**
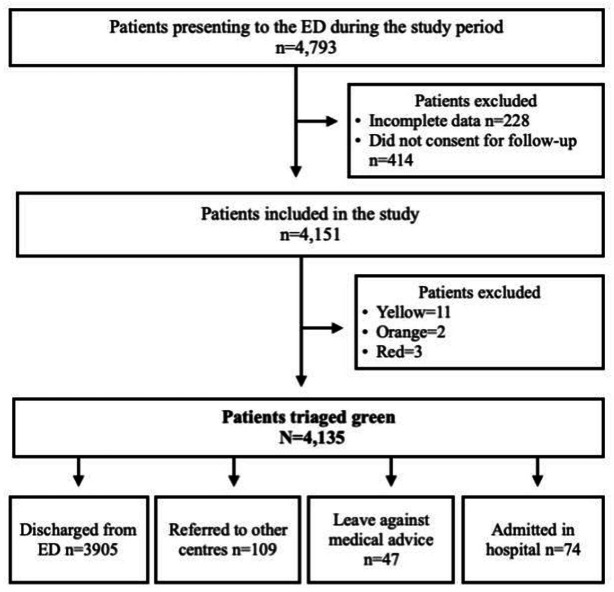
Study flow chart. ED, emergency department.

In the study, 4151 patients were included of which 4135 (99.6%) patients were triaged green by CTT (figure 1).

### Profile of patients triaged green

The mean age of patients was 32.8±13.1 years with 3172 (77%) males. Notably, of all patients triaged green, 10/4135 (0.24%) patients presented with moderate to severe GCS and 0.3% of patients did not have an AVPU of alert. The majority of patients (97%) triaged green presented to the study site directly without a primary care hospital referral.

**Figure 2:**
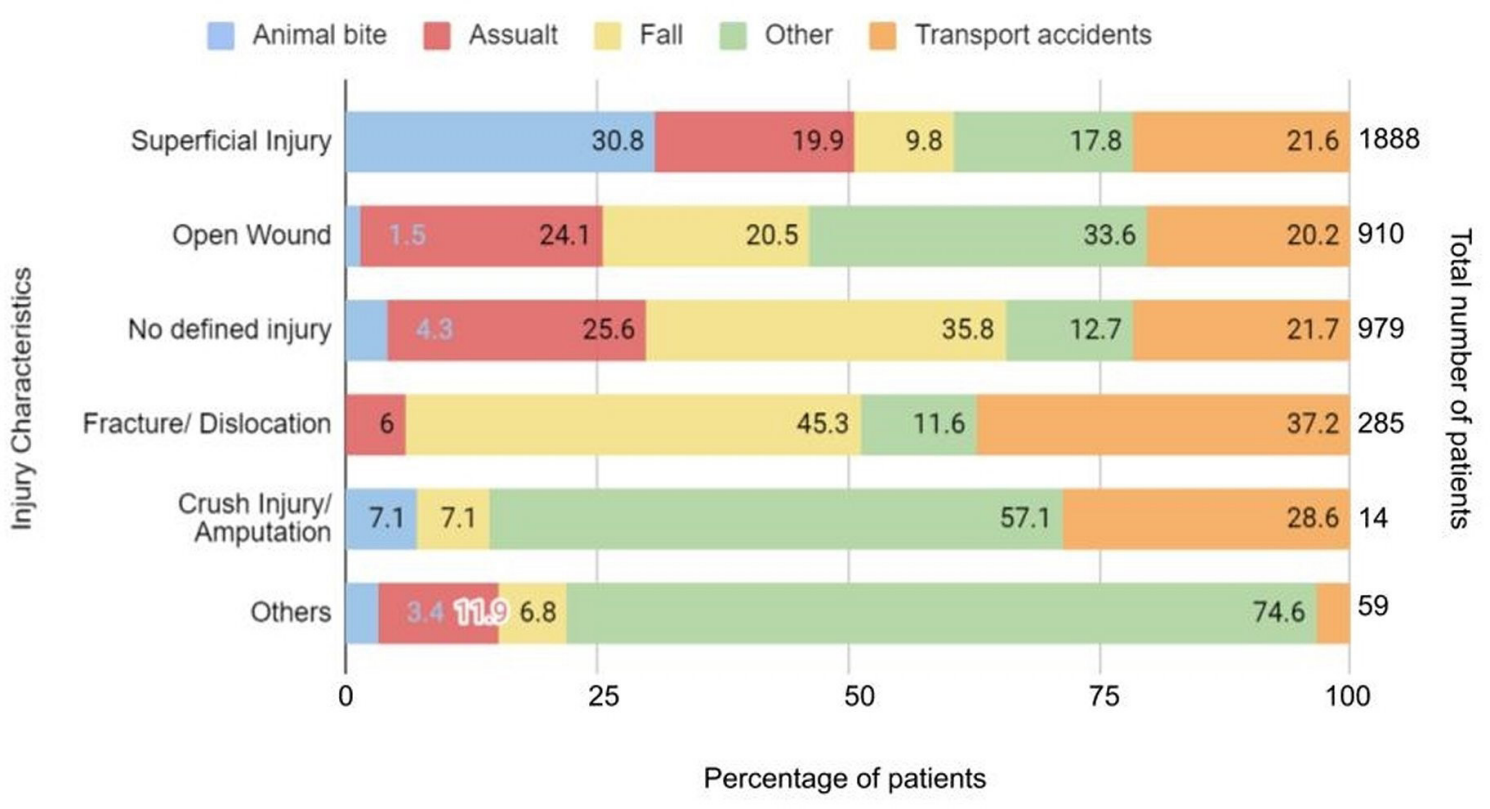
Percentage distribution of different injury mechanisms among injury types (N=4135).

Of the total patients triaged green by CTT, 46% of patients had only superficial injuries of which majority (30.8%) were due to animal bites. Further, 24% had no external injuries on examination. Among those referred to other centres, the most common types of injury identified were superficial injuries (34), followed by open wounds (27) and patients with no documented injury (19). The reasons for referral to other centres were not documented. As per ISS, 50.2% of patients had ‘mild’ and 0.4% had ‘moderate’ score and the remaining 49.5% patients had ‘no defined ISS’. Figure 2 shows the different injury types as per mechanism of injury in the study population. Among those that had a transport accident, 881/916 (96.17%) were patients who had a road traffic injury.

The ED disposition of all the patients is shown in figure 1. The median (IQR) LoS of those admitted to the hospital was 3 (13) days and seven patients required admission in the ICU. Most admitted patients 62/74 (83.8%) were successfully discharged from the hospital while three were transferred to other centres for further management. Further, there were eight patients who took LAMA and one who died during their hospital stay.

Follow-up at 30 days was successful for 3832/4135 (92.7%) of patients. Three patients died during the first 30 days. Of these patients, two had a GCS of <8 on initial evaluation. The rSATS triage of these patients were rYellow and rOrange.

### Evaluation of triage validity through rSATS

**Figure 3:**
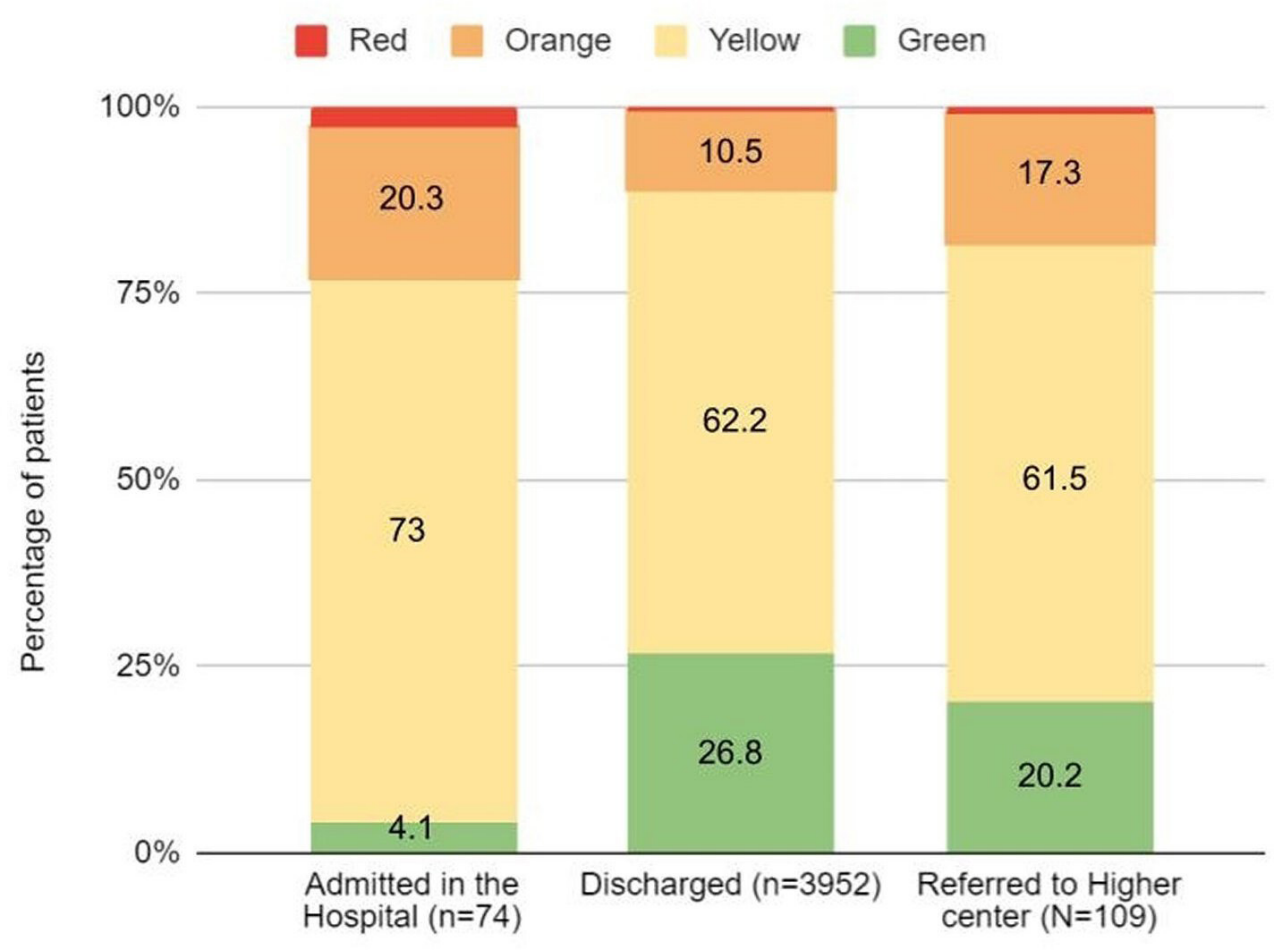
Patients’ disposition from ED as per retrospective triage using SATS (N=4135). ED, emergency department.

We found that of the total number of patients that were triaged green by CTT (N=4135), 24 patients were triaged rRed, 448 patients were triaged rOrange and 2579 patients were triaged rYellow as per rSATS indicating that 73.8% patients were undertriaged by CTT. Proportions of undertriage were higher during the night and afternoon (table 1A). Of these, most patients (97%) were found to have been undertriaged after assessing their physiological parameters from rTEWS while others due to the rSATScd as seen in table 1B. In figure 3, the disposition of these patients from the ED as per their rSATS is depicted. Notably, of the total three documented deaths, one occurred in a patient who was admitted in the hospital and triaged rOrange, and one in a patient transferred to a different centre triaged rYellow. Table 1C shows the rSATS of patients with fractures, dislocation and amputations.

**Table 1:** (A) Proportion of patients undertriaged as per the time of the day^24^ (B) Proportion of patients undertriaged as per rSATS (N=4135). (C) Injury characteristics as per the rSATS Triage category

(A) Patients undertriaged as per the time of the day^24^					
	Morning	Afternoon	Evening	Night	P value
n	756	1576	1155	648	
Undertriage	511 (67.6)	1185 (75.2)	858 (74.3)	497 (76.7)	<0.001

(B) Patients undertriaged as per rSATS (N=4135)				
rSATS	rGreen	rYellow	rOrange	rRed
n	1084	2579	448	24
rTEWS	1084 (100)	2513 (97.4)	433 (96.7)	19 (79.2)
rSATScd	0	66 (2.6)	15 (3.3)	5 (20.8)

(C) Injury characteristics as per the rSATS Triage category						
	rSATS	rGreen	rYellow	rOrange	rRed	P value
n		1084	2579	448	24	
	Amputation/crush injury	0 (0.0)	13 (0.5)	1 (0.2)	0 (0.0)	<0.001
	Fracture/dislocation	0 (0.0)	243 (9.4)	41 (9.2)	1 (4.2)	
Injury type	Others	9 (0.8)	36 (1.4)	9 (2.0)	5 (20.8)	
	Open wound	204 (18.8)	575 (22.3)	123 (27.5)	8 (33.3)	
	Superficial injury	635 (58.6)	1074 (41.6)	174 (38.8)	5 (20.8)	
	No defined injury	236 (21.8)	638 (24.7)	100 (22.3)	5 (20.8)	

rSATS, retrospectively used South African Triage Score; rSATScd, rSATS clinical discriminators; TEWS, Triage Early Warning Score.

## Discussion

### Main findings

Blunt trauma was seen as the most common type of injury.^24^ Transport accidents were the predominant mechanism of injury and 77% patients were males. Most patients had mild ISS (50.2%) and only about 0.4% patients had moderate ISS with no patients in the severe and profound ISS category. Most patients presented with seemingly superficial injuries. The presentation of 15.5% of patients with animal bites was unique to our setting. These patients mainly presented for vaccinations following animal bites more frequently than for the treatment of bite injuries.

This study shows that approximately three-quarters (74%) of patients triaged green by CTT were undertriaged when compared with rSATS. Out of 4135 patients triaged green by CTT only 1084/4135 (26.2%) were triaged green according to the rSATS. Most of these patients (97%) were coming in as direct arrival to this secondary-care hospital. According to CTT, patients were triaged green even with GCS moderate to severe (10/4135, 0.24%) and 0.3% of patients did not have an AVPU of alert and were still triaged green.

In addition to the high proportion of undertriage ascertained by rTEWS, of those admitted, seven required admissions to the ICU indicating they may have required urgent management for their condition. On a closer look at the physiological parameters of the three patients found dead on 30-day follow-up, it was seen that two of them were under triaged on initial evaluation as they had a GCS<8.

### Interpretation and clinical relevance

Among the green triaged patients, there were patients whose physiological parameters indicated that they required urgent attention although the proportion of these patients is relatively low compared with our sample size. These findings emphasise the need for an ED triage and an effective referral system based on on-scene triage. In addition, they highlight the efficacy of physiological scores such as TEWS, a component of SATS, in triaging patients accurately and the need to include GCS assessment for all patients presenting to the ED. Reasons for these patients being undertriaged must be explored extensively to enhance healthcare delivery in the EDs. Although the reasons for this undertriage are multifactorial, in this case, the lack of appropriate training or standard, uniform protocol for patient management in the ED to quickly identify these patients among those that have normal physiological parameters is most evident. The other factor, overcrowding of the ED with limitation of resources, may also lead to inadequate trauma care.^25^

### Strengths

This is a prospective cohort study, with vital signs recorded by a dedicated research officer, documenting the profile of green triaged patients from a public secondary care hospital in an urban Low- and Middle- Income Country (LMIC) setting conducted over a period of 3 years. Triage validity was assessed using a standardised and validated triage scoring system (SATS) that included both physiological parameters and injury characteristics of the patients.

### Limitations

First, the study provides data from a single secondary care centre, results of which may not be generalisable to other secondary care hospitals or other Indian healthcare settings. Second, to ensure feasibility, data of only 10 consecutive patients were collected in each shift. Third, we did not have data on the number of clinicians that participated in the triaging process or how they acquired knowledge and skills to triage patients. Lastly, 30-day mortality was missing for some patients while we have none on morbidity. This is a limiting factor towards assessing the morbidity gains.

## Conclusion

Three-fourths (74%) of the patients triaged green by clinicians in a secondary care hospital in Mumbai were undertriaged when analysed using rSATS. This highlights the need for implementation and evaluation of trauma triage training, involving systems that rely on presenting physiological parameters, for clinicians, nurses and other paramedical staff in the EDs. In addition, direct admissions of the non-urgent patients to this secondary care hospital warrants strengthening the referral systems to avoid overcrowding of the Indian EDs.

## Supplementary Material

Author's manuscript

## Data Availability

Data are available on reasonable request. Please write to the corresponding author for data request.
