# Supplementary material for: Profile and triage validity of trauma patients triaged green: a prospective cohort study from a secondary care hospital in India
Source: BMJ Open. 2023 May 8;13(5):e065036. doi: 10.1136/bmjopen-2022-065036 (PMC10173999; doi:10.1136/bmjopen-2022-065036)
Supplement: Supplementary data [file bmjopen-2022-065036supp002.pdf]

BMJ Publishing Group Limited (BMJ) disclaims all liability and responsibility arising from any reliance placed on this supplemental material which has been supplied by the author(s)

Supplementary material

Table of excluded International Classification of Diseases version 10 (ICD-10) codes

ICD-10 code	Short description	Full description
W65	Other external causes	Drowning and submersion while in bath-tub
W67	Other external causes	Drowning and submersion while in swimming-pool
W68	Other external causes	Drowning and submersion following fall into swimming-pool
W69	Other external causes	Drowning and submersion while in natural water
W70	Other external causes	Drowning and submersion following fall into natural water
W73	Other external causes	Other specified drowning and submersion
W74	Other external causes	Unspecified drowning and submersion
W78	Other external causes	Inhalation of gastric contents
W79	Other external causes	Inhalation and ingestion of food causing obstruction of respiratory tract
W80	Other external causes	Inhalation and ingestion of other objects causing obstruction of respiratory tract
W81	Other external causes	Confined to or trapped in a low-oxygen environment
W83	Other external causes	Other specified threats to breathing

W84	Other external causes	Unspecified threat to breathing
X20	Other external causes	Contact with venomous snakes and lizards

ICD-10 code	Short description	Full description
X21	Other external causes	Contact with venomous spiders
X22	Other external causes	Contact with scorpions
X23	Other external causes	Contact with hornets, wasps and bees
X24	Other external causes	Contact with centipedes and venomous millipedes (tropical)
X25	Other external causes	Contact with other venomous arthropods
X26	Other external causes	Contact with venomous marine animals and plants
X27	Other external causes	Contact with other specified venomous animals
X28	Other external causes	Contact with other specified venomous plants
X29	Other external causes	Contact with unspecified venomous animal or plant
X40	Other external causes	Accidental poisoning by and exposure to nonopioid analgesics, antipyretics and antirheumatics
X41	Other external causes	Accidental poisoning by and exposure to antiepileptic, sedative-hypnotic, antiparkinsonism and psychotropic drugs, not elsewhere classified
X42	Other external causes	Accidental poisoning by and exposure to narcotics and psychodysleptics [hallucinogens], not elsewhere classified

X43	Other external causes	Accidental poisoning by and exposure to other drugs acting on the autonomic nervous system
X44	Other external causes	Accidental poisoning by and exposure to other and unspecified drugs, medicaments and biological substances
X45	Other external causes	Accidental poisoning by and exposure to alcohol
X46	Other external causes	Accidental poisoning by and exposure to organic solvents and halogenated hydrocarbons and their vapours

ICD-10 code	Short description	Full description
X47	Other external causes	Accidental poisoning by and exposure to other gases and vapours
X48	Other external causes	Accidental poisoning by and exposure to pesticides
X49	Other external causes	Accidental poisoning by and exposure to other and unspecified chemicals and noxious substances
X50	Other external causes	Overexertion and strenuous or repetitive movements
X51	Other external causes	Travel and motion
X52	Other external causes	Prolonged stay in weightless environment
X53	Other external causes	Lack of food
X54	Other external causes	Lack of water
X57	Other external causes	Unspecified privation
X60	Intentional self-harm	Intentional self-poisoning by and exposure to nonopioid analgesics, antipyretics and antirheumatics

X61	Intentional self-harm	Intentional self-poisoning by and exposure to antiepileptic, sedative-hypnotic, antiparkinsonism and psychotropic drugs, not elsewhere classified
X62	Intentional self-harm	Intentional self-poisoning by and exposure to narcotics and psychodysleptics [hallucinogens], not elsewhere classified
X63	Intentional self-harm	Intentional self-poisoning by and exposure to other drugs acting on the autonomic nervous system
X64	Intentional self-harm	Intentional self-poisoning by and exposure to other and unspecified drugs, medicaments and biological substances
X65	Intentional self-harm	Intentional self-poisoning by and exposure to alcohol
X66	Intentional self-harm	Intentional self-poisoning by and exposure to organic solvents and halogenated hydrocarbons and their vapours
ICD-10 code	Short description	Full description
X67	Intentional self-harm	Intentional self-poisoning by and exposure to other gases and vapours
X68	Intentional self-harm	Intentional self-poisoning by and exposure to pesticides
X69	Intentional self-harm	Intentional self-poisoning by and exposure to other and unspecified chemicals and noxious substances
X85	Assault	Assault by drugs, medicaments and biological substances
X88	Assault	Assault by gases and vapours
X89	Assault	Assault by other specified chemicals and noxious substances
X90	Assault	Assault by unspecified chemical or noxious substance
Y06	Assault	Neglect and abandonment
Y060	Assault	Neglect and abandonment by spouse or partner

Y061	Assault	Neglect and abandonment by parent
Y062	Assault	Neglect and abandonment by acquaintance or friend
Y068	Assault	Neglect and abandonment by other specified persons
Y069	Assault	Neglect and abandonment by unspecified person
Y07	Assault	Other maltreatment
Y070	Assault	Other maltreatment by spouse or partner
Y071	Assault	Other maltreatment by parent
Y072	Assault	Other maltreatment by acquaintance or friend
Y073	Assault	Other maltreatment by official authorities
Y078	Assault	Other maltreatment by other specified persons
Y079	Assault	Other maltreatment by unspecified person
Y10	Event of undetermined intent	Poisoning by and exposure to nonopioid analgesics, antipyretics and antirheumatics, undetermined intent
ICD-10 code	Short description	Full description
Y11	Event of undetermined intent	Poisoning by and exposure to antiepileptic, sedative-hypnotic, antiparkinsonism and psychotropic drugs, not elsewhere classified, undetermined intent
Y12	Event of undetermined intent	Poisoning by and exposure to narcotics and psychodysleptics [hallucinogens], not elsewhere classified, undetermined intent
Y13	Event of undetermined intent	Poisoning by and exposure to other drugs acting on the autonomic nervous system, undetermined intent
Y14	Event of undetermined intent	Poisoning by and exposure to other and unspecified drugs, medicaments and biological substances, undetermined intent

Y15	Event of undetermined intent	Poisoning by and exposure to alcohol, undetermined intent
Y16	Event of undetermined intent	Poisoning by and exposure to organic solvents and halogenated hydrocarbons and their vapours, undetermined intent
Y17	Event of undetermined intent	Poisoning by and exposure to other gases and vapours, undetermined intent
Y18	Event of undetermined intent	Poisoning by and exposure to pesticides, undetermined intent
Y19	Event of undetermined intent	Poisoning by and exposure to other and unspecified chemicals and noxious substances, undetermined intent
Y352	Legal intervention and operations of war	Legal intervention involving gas
Y355	Legal intervention and operations of war	Legal execution
