# Supplementary material for: Profile and triage validity of trauma patients triaged green: a prospective cohort study from a secondary care hospital in India
Source: BMJ Open. 2023 May 8;13(5):e065036. doi: 10.1136/bmjopen-2022-065036 (PMC10173999; doi:10.1136/bmjopen-2022-065036)
Supplement: Supplementary data [file bmjopen-2022-065036supp001.pdf]

Table 1: Physiological and injury characteristics of patients triaged green (N=4135)

**Demographics**	
Mechanism of Injury (%)	
Transport accidents	916 (22.2)
Assault	870 (21)
Fall	856 (20.7)
Other	852 (20.6)
Animal bite	641 (15.5)
Transfer status	
Direct	4006 (97)
Transferred	129 (3)
**Vitals**	
AVPU (%)	
Unresponsive	3 (0.1)
Pain	6 (0.1)
Verbal	3 (0.1)
Alert	4123 (99.7)
GCS (mean (SD))	14.98 (0.4)
GCS (%)	
Mild	4125 (99.8)
Moderate	4 (0.1)
Severe	6 (0.1)
Systolic blood pressure (mean (SD))	128.05 (18.9)
Diastolic blood pressure (mean (SD))	84.34 (13.3)
Heart rate (mean (SD))	88.88 (17)
Oxygen saturation (mean (SD))	97.79 (2.2)
Respiratory rate (mean (SD))	22.63 (3.7)
Need for oxygen support (%)	
Not on oxygen support	4135 (100.0)
RTS (mean (SD))	7.99 (0.13)
**Injury characteristics**	
Type of Injury (%)	
Blunt	4075 (98.5)

Penetrating	56 (1.4)
Blunt & penetrating	4 (0.1)
Number of serious injury (%)*	
No serious injury	4112 (99.4)
Single	21 (0.5)
Multiple	2 (0.0)
ISS (%)	
No defined ISS	2048 (49.5)
Mild	2072 (50.1)
Moderate	15 (0.4)
Severe	0 (0.0)
Profound	0 (0.0)

*A serious injury was defined as an injury that warrants hospitalisation.(1)

Table 2A: **The Cape Triage Score depicting the TEWS** (2)

	Adult Triage Score						
	3	2	1	0	1	2	3
Mobility				Walking	With help	Stretcher/ Immobile	
RR		Less than 9		9-14	15-20	21-29	More than 29
HR		Less than 41	41-50	51-100	101-110	111-129	More than 129
SBP	Less than 71	71-80	81-100	101-199		More than 199	
Temp.		Less than 35		35-38.4		38.5 or more	
AVPU				Alert	Reacts to Voice	Reacts to Pain	Unresponsive
Trauma				No	Yes		
**Over 12 years / taller than 150 cm**							

Table 2B: The Cape Triage Score depicting the SATS color code (2)

Colour	Red	Orange	Yellow	Green
TEWS	7 or more	5 - 6	3-4	0-2
Target time to treat	Immediate	Less than 10 min	Less than 60 min	Less than 240 min
Mechanism of injury		High energy transfer		
Presentation		Shortness of breath - acute		All other patients
		Coughing blood		
		Chest pain		
		Haemorrhage uncontrolled	Haemorrhage - controlled	
	Seizure - current	Seizure-post ictal		
		Focal neurology - acute		
		Level of consciousness reduced		
		Psychosis/aggression		
		Threatened limb		
		Dislocation - other joint	Dislocation - finger or toe	
		Fracture - compound	Fracture - closed	
		Burn over 20%	Burn - other	
		Burn - electrical		
	Burn-face/ inhalation	Burn-circumferential		

		Burn-chemical		
		Poisoning/overdose	Abdominal pain	
	Hypoglycemia glucose less than 3	Diabetic - glucose over 11 & ketonuria	Diabetic - glucose over 17 (no ketonuria)	
		Vomiting - fresh blood	Vomiting - persistent	
		Pregnancy and abdominal trauma or pain	Pregnancy and trauma	
			Pregnancy and PV bleed	
Pain		Severe	Moderate	Mild
Senior health care professional's discretion				

References

1. Gardner A, Forson PK, Oduro G, Stewart B, Dike N, Glover P, et al. Diagnostic accuracy of the Kampala Trauma Score using estimated Abbreviated Injury Scale scores and physician opinion. Injury [Internet]. 2017 Jan 1 [cited 2021 Aug 23];48(1):177–83. Available from: https://pubmed.ncbi.nlm.nih.gov/27908493/

2. Rominski S, Bell SA, Oduro G, Ampong P, Oteng R, Donkor P. The implementation of the South African Triage Score (SATS) in an urban teaching hospital, Ghana. African journal of emergency medicine : Revue africaine de la medecine d'urgence [Internet]. 2014 [cited 2021 Aug 23];4(2):71–5. Available from: https://pubmed.ncbi.nlm.nih.gov/28344927/
