# Supplementary material for: Profile and triage validity of trauma patients triaged green: a prospective cohort study from a secondary care hospital in India
Source: BMJ Open. 2023 May 8;13(5):e065036. doi: 10.1136/bmjopen-2022-065036 (PMC10173999; doi:10.1136/bmjopen-2022-065036)
Supplement: Author's manuscript [file bmjopen-2022-065036.draft_revisions.pdf]

BMJ Open is committed to open peer review. As part of this commitment we make the peer review history of every article we publish publicly available.

When an article is published we post the peer reviewers' comments and the authors' responses online. We also post the versions of the paper that were used during peer review. These are the versions that the peer review comments apply to.

The versions of the paper that follow are the versions that were submitted during the peer review process. They are not the versions of record or the final published versions. They should not be cited or distributed as the published version of this manuscript.

BMJ Open is an open access journal and the full, final, typeset and author-corrected version of record of the manuscript is available on our site with no access controls, subscription charges or pay-per-view fees (http://bmjopen.bmj.com).

If you have any questions on BMJ Open's open peer review process please email info.bmjopen@bmj.com

Profile and Triage Appropriateness of Trauma Patients Triaged Green: A Prospective Cohort Study from a Secondary Care Hospital in India

Journal:	*BMJ Open*
Manuscript ID	bmjopen-2022-065036
Article Type:	Original research
Date Submitted by the Author:	06-Jun-2022
Complete List of Authors:	Anthony, Aroke; Doctors For You<br>Dutta, Rohini; - World Health Organization Collaborating Center for Research in Surgical Care Delivery in Low-and-Middle Income Countries; - Christian Medical College and Hospital,<br>Sarang, Bhakti; World Health Organization Collaborating Centre on Research in Surgical Care Delivery in Low-Middle-Income Countries; Terna Medical College & Hospital, Department of Surgery<br>David, Siddarth; Karolinska Institutet, Department of Global Public Health<br>O'Reilly, Gerard; Monash University, Emergency medicine<br>Raykar, Nakul; Harvard Medical School, Department of Global Health and Social Medicine; Brigham and Women's Hospital, Emergency Surgery<br>Khajanchi, Monty; Seth Gowardhandas Sunderdas Medical College and King Edward Memorial Hospital, Department of Surgery<br>Attergrim, Jonatan; Karolinska Institutet<br>Soni, Kapil; All India Institute of Medical Sciences, Critical Care<br>Sharma, Naveen; All India Institute of Medical Sciences, Surgery<br>Mohan, Monali; WHO Collaboration Centre for Research in Surgical Care Delivery in LMICs, Mumbai, India,<br>Gadgil, Anita; World Health Organization Collaborating Center for Research in Surgical Care Delivery in Low-and-Middle Income Countries<br>Roy, Nobhojit; World Health Organization Collaborating Center for Research in Surgical Care Delivery in Low-and-Middle Income Countries<br>Gerdin Wärnberg, Martin ; Karolinska Institutet, Department of Global Public Health; Karolinska University Hospital
Keywords:	TRAUMA MANAGEMENT, ACCIDENT & EMERGENCY MEDICINE, Trauma management < ORTHOPAEDIC & TRAUMA SURGERY

I, the Submitting Author has the right to grant and does grant on behalf of all authors of the Work (as defined in the below author licence), an exclusive licence and/or a non-exclusive licence for contributions from authors who are: i) UK Crown employees; ii) where BMJ has agreed a CC-BY licence shall apply, and/or iii) in accordance with the terms applicable for US Federal Government officers or employees acting as part of their official duties; on a worldwide, perpetual, irrevocable, royalty-free basis to BMJ Publishing Group Ltd ("BMJ") its licensees and where the relevant Journal is co-owned by BMJ to the co-owners of the Journal, to publish the Work in this journal and any other BMJ products and to exploit all rights, as set out in our licence.

The Submitting Author accepts and understands that any supply made under these terms is made by BMJ to the Submitting Author unless you are acting as an employee on behalf of your employer or a postgraduate student of an affiliated institution which is paying any applicable article publishing charge ("APC") for Open Access articles. Where the Submitting Author wishes to make the Work available on an Open Access basis (and intends to pay the relevant APC), the terms of reuse of such Open Access shall be governed by a Creative Commons licence – details of these licences and which Creative Commons licence will apply to this Work are set out in our licence referred to above.

Other than as permitted in any relevant BMJ Author's Self Archiving Policies, I confirm this Work has not been accepted for publication elsewhere, is not being considered for publication elsewhere and does not duplicate material already published. I confirm all authors consent to publication of this Work and authorise the granting of this licence.

For peer review only

Profile and Triage Appropriateness of Trauma Patients Triaged Green: A Prospective Cohort Study from a Secondary Care Hospital in India

Aroke Anna Anthony,[1,2] Rohini Dutta,[2,3] Bhakti Sarang,[2,4] Siddarth David,[5] Gerard O'Reilly,[6,7,8] Nakul Raykar,[9,10,11] Monty Khajanchi,[2,12] Jonatan Attergrim,[13] Kapil Dev Soni,[14] Naveen Sharma,[15] Monali Mohan,[2] Anita Gadgil,[2] Nobhojit Roy,[2,5,7] Martin Gerdin Wärnberg[5,13]

[1] Doctors For You, Mumbai, India

[2] Trauma Research Group, World Health Organization Collaborating Centre on Research in Surgical Care Delivery in Low-Middle-Income Countries, Mumbai, India

[3] MBBS, Christian Medical College and Hospital, Ludhiana, India

[4] Department of Surgery, Terna Medical College & Hospital, New Mumbai, India

[5] Department of Global Public Health, Karolinska Institutet, Stockholm, Sweden

[6] Epidemiology & Biostatistics, National Trauma Research Institute, The Alfred, Melbourne, Australia

[7] School of Public Health & Preventive Medicine, Monash University, Melbourne, Australia

[8] Global programs, Emergency & Trauma Centre, The Alfred, Melbourne, Australia

[9] Program in Global Surgery and Social Change, Harvard Medical School, Boston, USA

[10] Trauma, Emergency Surgery & Surgical Critical Care, Brigham and Women's Hospital, Boston, MA, USA

[11] Center for Surgery and Public Health, Brigham and Women's Hospital, Boston, MA, USA

[12] Department of Surgery, Seth.G.S. Medical College & K.E.M. Hospital, Parel, Mumbai, India

[13] Function Perioperative Medicine and Intensive Care, Karolinska University Hospital, Stockholm, Sweden

[14] Critical & Intensive Care, JPN Apex Trauma Centre, AIIMS, New Delhi, India

[15] Department of Surgery, All India Institute of Medical Sciences, Jodhpur, India

Corresponding Author: Martin Gerdin Wärnberg

Department of Global Public Health, Karolinska Institutet, Stockholm, Sweden; Function Perioperative Medicine and Intensive Care, Karolinska University Hospital, Stockholm, Sweden.

Address: Department of Global Public Health
Karolinska Institutet
171 77 Stockholm

Email: martin.gerdin@ki.se

Phone Number: +46 708539598

For peer review only - http://bmjopen.bmj.com/site/about/guidelines.xhtml

Short title: Profile and Triage Appropriateness of Trauma Patients Triaged Green in a Secondary Care Hospital in India

Word Count (Abstract): 239 words

Word Count (Manuscript): 4472 words

For peer review only

ABSTRACT

Objective: To evaluate the profile of non-urgent patients triaged 'green', as part of a triage-trial in the Emergency Department (ED) of a secondary-care hospital in India. The secondary aim was to validate our findings with the Cape Triage Score (CTS).

Design: Prospective cohort study

Setting: A secondary care-hospital in Mumbai, India.

Participants: Patients aged 18 years and above with a history of trauma defined as having any of the external causes of morbidity and mortality listed in block V01-Y36, chapter XX of the International Classification of Disease version 10 (ICD-10) codebook, triaged green between July 2016 to November 2019.

Outcome: Primary outcome measures were mortality within 24-hours, 30-days, and 6-months.

Results: We included 4135 trauma patients triaged green. The mean age of patients was 32.8 (±13.1) years, and 77% were males. The median (IQR) length of stay of admitted patients was 3 (13) days. Half the patients had a mild Injury Severity Score (3-8), with the majority of injuries being blunt (98%). Of the patients triaged green by clinicians, three-quarters (74%)were undertriaged on cross validating with CTS. On telephonic follow-up two patients were reported dead whereas one died while admitted in-hospital.

Conclusion: Our study highlights the need for implementation and evaluation of trauma triage training for the in-hospital first responders (clinicians, nurses and other paramedical staff) in the EDs.

Ethical clearance: Ethics committee approval for TTRIS was obtained from the ethics and scientific committee of KBBH (KBBH, HO/4982/KBB,12/08/2016).

For peer review only

Strengths and limitations of this study:

- It is the first study from a secondary care hospital in India that provides the profile and outcomes of patients triaged green.
- The study provides robust data from one of the largest studies done in a secondary care hospital in a LMIC setting.
- The study provides data from a single secondary care hospital in Mumbai. Therefore, the results cannot be generalised to other Indian hospitals due to hospital bias.
- Data of only the first 10 consecutive patients were collected each shift during the study period.
- Data on morbidity and from autopsy reports were unavailable to determine the morbidity outcomes and exact cause of death.

For peer review only

For peer review only - http://bmjopen.bmj.com/site/about/guidelines.xhtml

INTRODUCTION

Globally, each year, 4.5 million people die from trauma, with India contributing to 20% of this burden.(1) Trauma represents the second most common cause of death after age five in India.(2) Furthermore, India also has the highest rank in the number of deaths attributable to road traffic-related deaths in the world.(3) Approximately 50% of trauma deaths occur in the hospitals, highlighting the need for strengthening in-hospital care.(4) Trauma care is highly time-sensitive, and the early identification of injuries is important for survival.(5) Establishment of hospital triage systems can ensure that critically ill patients are identified and receive care promptly.(6) For this purpose, several triage scores are used across different countries and hospital settings.(7,8)

In India, the high population density, poorly developed prehospital care and a lack of appropriate referral systems leads to overcrowding in the emergency departments (EDs).(9–12) Most EDs lack triage protocols and the level of emergency patient care is decided by clinicians who are not trained specifically in trauma care.(13,14) Trauma management trainings are also not incorporated as a separate subject in the medical training of clinicians. Inappropriate triage is known to have contributed to a surge in non-urgent patients, exacerbating the problem of overcrowding in the ED.(14–17) This may be due to prioritising the evaluation of patients with low urgency, which results in the diversion of workforce and resources from serious patients requiring immediate care. Nonetheless, not evaluating these patients could result in missed injuries and poor outcomes.

In our study, clinicians at a triage-naive ED were introduced to a triage-trial, as part of a multicentre triage project which compared prediction models for triage in adult trauma patients presenting to various emergency departments across India.(15) The patients were designated one of the four trauma triage categories by clinicians, based on their understanding of trauma triage; into red, orange, yellow, green, with red and green denoting the most and least urgent patient status

For peer review only - http://bmjopen.bmj.com/site/about/guidelines.xhtml

respectively. We aimed to evaluate the profile of the non-urgent patients who were triaged green by clinicians and the validity of this category in comparison to the Cape Triage Score (CTS). CTS is a mixed score based on physiological parameters and the pathology of the patient and has been effectively used at various settings in South Africa and Low- and Middle-Income Countries (LMIC) since 2006.(18) It is a comprehensive triage score with a low undertriage rate capable of predicting patient disposition.(19)

METHODOLOGY

Study Design

This single-centre prospective cohort study is part of the Trauma Triage Study in India (TTRIS) which compares prediction models for triage in adult trauma patients presenting to various emergency departments across India. Data was collected in the study site from July 2016 to November 2019.

Study Setting

The study site was the ED of Khurshedji Behramji Bhabha Hospital (KBBH), a 436 bedded regional secondary healthcare centre located in Mumbai, India, catering to approximately 350 patients each day in the ED. It is a secondary-care public hospital with free or nominal fees, providing access to low socio-economic groups and receives patients from across the city. At KBBH, trauma care is imparted as a subspeciality along with medical, surgical, and obstetric care. The hospital has an intensive care facility but there is no neurosurgery department, so patients in need of neurosurgical management are referred to tertiary care centres. Plain radiography and ultrasonography are available round the clock; however computerized tomography (CT) is only available in-house from 7am to 6pm. The patients arriving at the ED are first seen by a casualty

For peer review only - http://bmjopen.bmj.com/site/about/guidelines.xhtml

medical officer (CMO) largely on a first-come, first-served basis without a formalised system of triaging patients at the ED.

Clinician Triage

As part of data collection of TTRIS, the triage-naive clinicians were informed about the trauma triage categories, without provision to any formal tool or training about the same. The clinicians involved have a minimum of 2 years clinical experience, however, they are neither trained in trauma care as a speciality nor are they necessarily trained in trauma management courses like ATLS. Medical training of the clinicians include aspects such as triage systems in theory however not put in practise due to lack of formal triage protocols. After their initial on-arrival assessment of each patient, the research officers asked the clinicians to categorise the patient as per their understanding of how urgently the patient requires treatment into the aforementioned colour-coded triage groups; red, orange, yellow, green, with red and green denoting the most and least urgent patient status respectively,(15) henceforth referred to as the triage levels. The clinicians were allowed to use all available information that was extracted by them during initial routine assessment (such as wound assessment) along with patient vitals that were collected by the research officer for determining the urgency of treatment required and thereby the triage level. The triage levels were not used to determine treatment decisions in the ED as there was no formalised tool or protocol in place for assigning the triage and coupling it with patient management. The clinicians were individually informed about the aim and methodology used for the TTRIS study at the start of their respective posting at the ED, however, the clinicians were neither involved in the conception nor were they part of the research team analysing the results.

Participants

For peer review only - http://bmjopen.bmj.com/site/about/guidelines.xhtml

Inclusion Criteria: We included all the patients aged 18 years and above presenting to the KBBH ED with a history of trauma and triaged green by clinical triage on initial evaluation irrespective of their injury severity. A history of trauma was defined here as having any of the external causes of morbidity and mortality listed in block V01-Y36, chapter XX of the International Classification of Disease version 10 (ICD-10) online codebook, with some exclusions (see online supplementary material).(20) Only patients with above mentioned causes of trauma as their primary complaint were included. For example, patients with a history of fall due to dizziness were not included in the study.

Exclusion Criteria: Patients with one or more vital parameters missing among the variables used for analysis or who did not consent to follow-up were excluded from the analysis.

Source and methods of selection of participants and follow-up

The research officer at KBBH observed morning, evening and night shifts (6-hour observational shifts). These shifts were not aligned with the working hours of the clinical staff to reduce bias and accounting for shift fatigue of the clinicians. Data were collected from the first 10 consecutive patients only, irrespective of their triage, during each shift. Due to the large patient load and time and budgetary constraints of the project, data collection of only the first 10 patients was considered feasible for follow-up.

The research officer performed follow-up at 24-hours, 30-days and 6-months after arrival at the ED. The time frame of the study was chosen to ensure that all included patients had completed 6 months of follow-up to minimise the loss to follow-up. The follow-up was completed in-person or by telephone, depending on whether the patient was still hospitalised or if the patient had been discharged. The phone numbers of one or more contact persons, mostly relatives, were collected on enrolment and those people were contacted if the participant did not reply to the follow-up

For peer review only - http://bmjopen.bmj.com/site/about/guidelines.xhtml

telephone calls. The outcome was recorded as missing if neither the patient nor the relative were available for follow-up at the specified time-points.

Variables

The outcome measures were mortality within 24-hours, 30-days and 6-months.

Additionally, for each participant, age, sex, transfer status, time of injury, mechanism of injury, injury-related details, number of serious injuries, and the assigned informal triage category were collected. Physiological measures including systolic blood pressure (SBP), respiratory rate (RR), heart rate (HR), peripheral capillary oxygen saturation (SpO_2), Glasgow Coma Scale (GCS) and Alert Verbal Pain unresponsive scale (AVPU) were recorded.

A serious injury was defined as an injury that warrants hospitalisation.(21) GCS was categorised into no or mild traumatic brain injury (TBI) (13-15), moderate TBI (9-12), severe TBI (3-8).(22) ED-length of stay (ED-LoS) was calculated using the date and time of arrival and the date and time of discharge from the ED, either to be sent home or admitted in the hospital. Length of stay in the hospital (LoS) was calculated using the data and time of admission in the hospital to the data and time of discharge alive from the hospital, mortality, Leave against medical advice (LAMA) or abscond. Injury severity score (ISS) was allocated retrospectively with 'mild' (3-8), 'moderate' (9-15), 'severe' (16-25) and 'profound' (>25) categories. Patients for whom ISS could not be coded, for example when there were no recorded injuries, were assigned 'no defined ISS'.(23) The revised trauma score (RTS) was computed and categorised as RTS < 4 and RTS > 4.(23,24)

Injuries were recorded and coded using ICD-10 in the TTRIS dataset. Patients were divided into categories with respect to the most critical injury namely, crush injury, injury to internal organs, blood vessel injury, amputation, fracture, dislocation, burn, multiple injury, unspecified injury, open wound, superficial injury.(20) Injury characteristics of patients that presented to the ED with

For peer review only - http://bmjopen.bmj.com/site/about/guidelines.xhtml

no injuries were categorized as ‘no defined injury’. For patients with multiple injuries, the more critical one was considered for categorising patients as per injury.

Triage as per Cape Triage Score (CTS) System

The CTS has three versions, the adult version (those over 12 years of age or 150 cm in height), the child version (those between 3 - 12 years old or 95 - 150 cm) and the infant version (those less than 3 years of age or under 95 cm).(18) The adult CTS was used retrospectively to check for the appropriateness of the informal triage performed by the clinicians.(18) The physiological parameters were each scored against the adult Triage Early Warning Score (TEWS) scoring sheet, to calculate a total TEWS **(Table 1)**. Each patient was assigned a triage level that corresponds to the TEWS score as follows: 0-2 green, 3-4 yellow, 5-6 orange, 7 or more red as seen in **Table 2**. Each patient was further categorised into the four triage levels using the Cape Triage Group (CTG) list of discriminators, also known as the South African Triage Scale (SATS) colour code. If a patient was categorised into a higher level by the SATS colour code than the TEWS score, then the higher level was considered as the correct triage level. For example, if the SATS color code categorised a patient with closed fracture into yellow, the triage level assigned to the patient would be yellow even if the TEWS score categorised the patient into green. The physiological parameters are considered for assigning tirage and the discriminators are used as a safety net in case patients do not present with abnormal physiology.

For peer review only

Table 1: **The Cape Triage Score depicting the TEWS** (18)

Adult Triage Score

	3	2	1	0	1	2	3
Mobility				Walking	With help	Stretcher/ Immobile	
RR		Less than 9		9-14	15-20	21-29	More than 29
HR		Less than 41	41-50	51-100	101-110	111-129	More than 129

For peer review only - http://bmjopen.bmj.com/site/about/guidelines.xhtml

SBP	Less than 71	71-80	81-100	101-199		More than 199	
Temp.		Less than 35		35-38.4		38.5 or more	
AVPU				Alert	Reacts to Voice	Reacts to Pain	Unresponsive
Trauma				No	Yes		
**Over 12 years / taller than 150 cm**							

Table 2: The Cape Triage Score depicting the SATS color code (18)

Colour	Red	Orange	Yellow	Green
TEWS	7 or more	5 - 6	3-4	0-2
Target time to treat	Immediate	Less than 10 min	Less than 60 min	Less than 240 min
Mechanism of injury		High energy transfer		
Presentation		Shortness of breath - acute		All other patients
		Coughing blood		
		Chest pain		
		Haemorrhage uncontrolled	Haemorrhage - controlled	
	Seizure - current	Seizure-post ictal		
		Focal neurology - acute		
		Level of consciousness reduced		
		Psychosis/aggression		
		Threatened limb		
		Dislocation - other joint	Dislocation - finger or toe	
		Fracture - compound	Fracture - closed	
		Burn over 20%	Burn - other	
	Burn-face/ inhalation	Burn - electrical		
		Burn-circumferential		
		Burn-chemical		

For peer review only - http://bmjopen.bmj.com/site/about/guidelines.xhtml

		Poisoning/overdose	Abdominal pain	
	Hypoglycemia glucose less than 3	Diabetic - glucose over 11 & ketonuria	Diabetic - glucose over 17 (no ketonuria)	
		Vomiting - fresh blood	Vomiting - persistent	
		Pregnancy and abdominal trauma or pain	Pregnancy and trauma	
			Pregnancy and PV bleed	
Pain		Severe	Moderate	Mild
Senior health care professional's discretion				

For peer review only

Quality assurance

There were three layers of quality control. First, data was entered using a dedicated electronic data collection instrument with extensive logical checks and prompts for unlikely but possible values. Second, the collected data were reviewed on a weekly basis and discussed during weekly online conferences with all project officers and the project leads throughout the duration of the data collection period. Third, on-site quality control sessions were conducted every 3-4 months. During these sessions, a second research officer collected data alongside the research officer who worked at the ED. The quality-controlled data was then compared with the standard data.

Patient and Public Involvement

No patients were involved.

Data Analysis

Data analysis was performed using R version 4.04 statistical software.(25) Complete case analysis was performed to exclude all patients with missing data among the variables included for analysis. Descriptive statistics were generated for all variables. The results are presented as frequencies and

For peer review only - http://bmjopen.bmj.com/site/about/guidelines.xhtml

percentages for categorical variables, mean and standard deviation (SD) for continuous variables and median and inter quartile range (IQR) for variables with abnormal distribution, for example LoS. The patients triaged green as per the clinician triage were further triaged as per the CTS, using the TEWS and SATS colour code into red, orange, yellow and green. The number of patients triaged green by the CTS was divided by the number of patients triaged green as per clinician triage (4135), the resultant proportion minus one was considered as the proportion of patients mis-triaged.

RESULTS

In the study, 4151 patients were included of which 4135 (99.6%) patients were triaged green by the clinicians **(Figure 1)**. The mean age of patients was 32.8±13.1 years with 3172 (77%) males. **Table 3** shows the physiological parameters at the time of presentation and injury characteristics as per the ISS of the study population. Notably, of all patients triaged green, 10/4135 (0.24%) patients presented with moderate to severe GCS and 0.3% of patients did not have an AVPU of alert. Majority of patients triaged green (97%) presented to the study-site directly without a primary care hospital referral. Blunt injury (98.5%) was the most common injury presentation with penetrating injury found in only 1.4% of patients. The mean (SD) revised trauma score (RTS) of these patients triaged green was 8 (0.13) with all patients having an RTS > 4.

Table 3: Physiological and injury characteristics of patients triaged green (N=4135)

**Demographics**	
Mechanism of Injury (%)	
Transport accidents	916 (22.2)
Assault	870 (21)

For peer review only - http://bmjopen.bmj.com/site/about/guidelines.xhtml

Fall	856 (20.7)
Other	852 (20.6)
Animal bite	641 (15.5)
Transfer status	
Direct	4006 (97)
Transferred	129 (3)
**Vitals**	
AVPU (%)	
Unresponsive	3 (0.1)
Pain	6 (0.1)
Verbal	3 (0.1)
Alert	4123 (99.7)
GCS (mean (SD))	14.98 (0.4)
GCS (%)	
Mild	4125 (99.8)
Moderate	4 (0.1)
Severe	6 (0.1)
Systolic blood pressure (mean (SD))	128.05 (18.9)
Diastolic blood pressure (mean (SD))	84.34 (13.3)
Heart rate (mean (SD))	88.88 (17)
Oxygen saturation (mean (SD))	97.79 (2.2)
Respiratory rate (mean (SD))	22.63 (3.7)
Need for oxygen support (%)	
Not on oxygen support	4135 (100.0)
RTS (mean (SD))	7.99 (0.13)
**Injury characteristics**	
Type of Injury (%)	

For peer review only - http://bmjopen.bmj.com/site/about/guidelines.xhtml

Blunt	4075 (98.5)
Penetrating	56 (1.4)
Blunt & penetrating	4 (0.1)
Number of serious injury (%)	
No serious injury	4112 (99.4)
Single	21 (0.5)
Multiple	2 (0.0)
ISS (%)	
No defined ISS	2048 (49.5)
Mild	2072 (50.1)
Moderate	15 (0.4)
Severe	0 (0.0)
Profound	0 (0.0)

Injury Characteristics

Of the total patients triaged green by clinicians, 46% of patients had only superficial injuries of which majority (30.8%) were due to animal bites. Further, 24% had no history or evidence of injuries on examination. Among those referred to other centers, the most common types of injury identified were superficial injuries (34) followed by open wounds (27) and patients with no documented injury (19). The reasons for referral to other centres were not documented. As per ISS, 50.2% of patients had 'mild' and 0.4% had 'moderate' score and the remaining 49.5% patients had 'no defined ISS'. **Figure 2** shows the different injury types as per mechanism of injury in the study population. Amongst those that had a transport accident, 881/916 (96.17%) were patients who had a road traffic injury.

Patient Outcomes

For peer review only - http://bmjopen.bmj.com/site/about/guidelines.xhtml

The ED disposition of all the patients is shown in **Figure 1**. The median (IQR) length of stay (LoS) of those admitted to the hospital was 3 (13) days and seven patients required admission in the intensive care unit. Most admitted patients 62/74 (83.8%) were successfully discharged from the hospital while three were transferred to other centers for further management. Further, there were eight patients that left against medical advice and one who died during their hospital stay.

Patient Mortality

Follow up at 30 days was successful for 3832/4135 (92.7%) of patients. Three patients died during the first 30 days. The first patient who died while in-hospital was an 80-year-old woman, who arrived at the ED 7 days after injury following a fall at ground level. With a GCS of 7 on arrival, she was admitted to the ICU with an ED-LoS of 30 minutes. Documented injury of the patient was an old contused lacerated wound on the forehead and abrasion on arm. She died within 36 hours of admission. The patient was retrospectively triaged orange as per the CTS.

The second patient, a 60-year-old man with a GCS of 8 was triaged green by the clinician. The patient arrived at the ED within 45 minutes of injury due to a fall from height. The patient was transferred to another centre with an ED-LoS of 1 hour 50 minutes and was alive at 24-hour follow-up but not admitted at the transferred centre at the time of follow-up. He was reported dead at 30 days follow-up and the cause of death could not be deduced from the available data. The patient was retrospectively triaged yellow as per the CTS.

The third patient, an 80-year-old man arrived in the ED within an hour following injury due to fall (W18) with a complaint of pain in the hip. On arrival, the patient had a GCS of 15 and oxygen saturation of 98%. The radiological findings (X-ray chest and X-ray pelvis and both hips) were normal and he was discharged from the ED with an ED-LoS of less than 3 hours. On the 30-day follow-up, the patient had died. This patient was triaged yellow as per the CTS.

For peer review only - http://bmjopen.bmj.com/site/about/guidelines.xhtml

Follow up at 6 months was successful for 3597/4132 (87%) green-triaged patients. A 69 year old woman was reported dead. The patient arrived in the ED 6 days following a fall from the bed (W06) with a GCS of 15. The patient had no documented injuries and was discharged with an ED-LoS of 1 hour. The patient was retrospectively triaged yellow as per CTS. The cause of death could not be ascertained due to the non-availability of death records and autopsy findings.

Evaluation of triage appropriateness through retrospective Cape Triage Score (CTS)

We found that of the total number of patients that were triaged green by clinicians (N=4135), 24 patients were triaged red, 448 patients were triaged orange and 2579 patients were triaged yellow as per CTS indicating that 73.8% patients were mistriaged by the ED clinicians. Of these, most patients (97%) were found to have been mistriaged after assessing their physiological parameters from TEWS while others due to the SATS color code for discriminators as seen in **Table 4**. In **Figure 3** the disposition of these patients from the ED as per their CTS is depicted. Notably, of the total four documented deaths, one occurred in a patient who was admitted in the hospital and triaged orange as per CTS, and one in a patient transferred to a different centre triaged yellow as per CTS.

Table 4: Patients mistriaged as per CTS (N=4135)

	Green	Yellow	Orange	Red
**Cape Triage Score**	1084	2579	448	24
**Triage Early Warning Score**	1084	2513	433	19
**South African Triage Scale**	0	66	15	5

DISCUSSION

For peer review only - http://bmjopen.bmj.com/site/about/guidelines.xhtml

Our study revealed that approximately three quarters (74%) of patients informally triaged green were effectively mistriaged when compared to a validated triage system and as per CTS only 1084/4135 (26.2%) were triaged green. Of the patients triaged green, 94.4% were discharged from the ED, apparently indicating that these patients may be coming in with presentations that do not require hospital admission. Also, most of these patients (97%) were coming in as direct arrival to this secondary-care hospital. This emphasises the need of on-scene triage and an effective referral system in order to prevent overburdening of the EDs of the secondary and tertiary-care hospitals.

Blunt trauma was seen as the most common mechanism of injury. A similar trauma mechanism was noted in a pilot implementation of a trauma registry study from Pakistan.(26) Superficial injury was the most frequent presentation and the predominant mechanism of injury was found to be transport accidents followed by assault. These findings are similar to a study describing the profile of patients presenting to the ED of a general hospital, similar to our setting, in southern Ethiopia.(27) The presentation of 15.5% of patients with animal bites was unique to our setting. These patients mainly presented for vaccinations following animal bites more frequently than for the treatment of bite injuries.

Only 2.6% of green-triaged patients were referred to other centers for further management. This referral rate is low in comparison to a study from southern Ethiopia that reported a rate of 5.2%.(27) However, in our study, it is worth noting that most patients who were referred to higher centres only had superficial, open wounds, or no defined injuries. So, it is difficult to assume if these transfers were genuinely warranted or could have been managed in the same hospital. Additionally, transfers due to overcrowding during specific hours of the day, overwhelming the existing infrastructure, resources or manpower at that particular time, may be a possibility.

For peer review only - http://bmjopen.bmj.com/site/about/guidelines.xhtml

Overcrowding of the ED, with limitation of resources, seems to be an important factor for inadequate trauma care.(28) This makes triaging crucial which enables intensifying the efforts towards patients requiring immediate interventions and quick management and disposition of the less urgent patients. Physiological parameters are most frequently used by clinicians to triage patients in India owing to lack of access to investigations such as CT scan during all times of the day. These on-arrival parameters are also known to be the most effective in the case of low-resource settings in predicting patient outcomes.(28) Our study found that 10/4135 (0.24%) patients that reported GCS moderate to severe and 0.3% of patients did not have an AVPU of alert and were still triaged green. This indicates that among the green triaged patients with close to normal physiological parameters, there were patients that required urgent attention. Although the proportion of these patients is relatively low compared to our sample size, reasons for these patients being inappropriately triaged must be explored extensively to enhance healthcare delivery in Indian EDs. Although the reasons for mistriaging are multifactorial, in this case, the lack of appropriate training or standard, uniform protocol for patient management in the ED to quickly identify these patients among those that have normal physiological parameters is most evident. Additionally, these findings highlight the efficacy of physiological scores such as TEWS, a component of CTS in triaging patients accurately and the need to include GCS assessment for all patients presenting to the ED. Furthermore, vital signs-based prediction models have been found to be beneficial for busy resource-limited public hospitals in urban India, where access to imaging modalities is not available around-the-clock.(29)

This need for reinforcement of adequate formal triage training is strengthened by our finding that as per CTS, nearly three-quarters (74%) of patients were mistriaged green of which, most (97%) were ascertained by their Triage Early Warning Score (TEWS), that takes into account

For peer review only - http://bmjopen.bmj.com/site/about/guidelines.xhtml

physiological parameters. In addition, of those admitted, seven required admission to the ICU indicating they may have required urgent management for their condition. On a closer look at the triage category denoted by clinicians at the three patients found dead on 30-day follow-up, it was seen that two of them were under triaged on initial evaluation of physiological parameters as they had a GCS < 8. Further, in our study, retrospective triage using a physiological score reveals its benefits if implemented in a low-resource setting as seen with TEWS, which identified the majority of patients mistriaged (Table 4). Including appropriate triage training for CMO's when implementing reforms in trauma management is a key step towards improving outcomes in trauma patients. This requires prioritisation of meticulous evaluation of the initial vital parameters by the ED staff to reduce errors and improve outcomes, in addition to addressing other contributing factors such as the low clinician to patient ratio in Indian EDs.

This prospective cohort study is one of the largest studies done in an LMIC setting, conducted for 3.5 years that provides robust data from a secondary care hospital in Mumbai. The hospital serves patients directly presenting to the ED and also patients that are referred from primary and other secondary care centres. This is also the first study from India that provides an in-depth profile and outcomes of patients triaged green in a low-resource hospital setting. However, this study has limitations. Firstly, the study provides data from a single secondary care centre, results of which may not be generalisable to other secondary care hospitals or other Indian healthcare settings, due to hospital bias. Secondly, to ensure feasibility, data of only 10 consecutive patients were collected in each shift. Due to which we do not have data of all the patients coming to the ED. Thirdly, we did not have data from autopsy reports of individuals that died to ascertain their exact cause of death. Lastly, we have data only on mortality of the patients but no additional data documenting

For peer review only

the morbidity. This is a limiting factor towards assessing the morbidity gains after implementation of triage training.

CONCLUSION

Three-fourths (74%) of the patients triaged green by clinicians in a secondary care hospital in Mumbai were mistriaged when retrospectively analysed using CTS. This highlights the need for implementation and evaluation of trauma triage training for the in-hospital first responders (clinicians, nurses and other paramedical staff) in the EDs. Also, direct admissions of the non-urgent patients to this secondary-care hospital warrants strengthening the referral systems to avoid overcrowding of the Indian EDs.

LIST OF ABBREVIATIONS

Emergency department (ED), Cape Triage Score (CTS), Low- and Middle-Income Country (LMIC), Trauma Triage Study in India (TTRIS), Khurshedji Behramji Bhabha Hospital (KBBH), Computerized Tomography (CT), Casualty Medical Officer (CMO), International Classification of Disease version 10 (ICD-10), Injury Severity Score (ISS), systolic blood pressure (SBP), respiratory rate (RR), heart rate (HR), peripheral capillary oxygen saturation (SpO2), Glasgow Coma Scale (GCS), Alert Verbal Pain unresponsive scale (AVPU), Traumatic Brain Injury (TBI), ED-length of stay (ED-LoS), Length of stay in the hospital (LoS), Leave against medical advice (LAMA), Revised Trauma Score (RTS), Triage Early Warning Score (TEWS), South African Triage Scale (SATS), Standard deviation (SD), Inter quartile range (IQR)

DECLARATIONS

For peer review only - http://bmjopen.bmj.com/site/about/guidelines.xhtml

Ethical clearance

Ethics committee approval for TTRIS was obtained from the ethics and scientific committee of KBBH (KBBH, HO/4982/KBB,12/08/2016). Informed consent for follow-up was taken from patients at the time of discharge from the hospital. In case the patient was unconscious, consent was obtained from a family member or the patient's legally acceptable representative.

Consent for publication

Not Applicable

Availability of data and materials

The data are available to whoever wants them by emailing the corresponding author. They can write their aims and objectives, and then, the authors can decide if that study can be done without duplication of the work.

Competing Interests

There is no conflict of interest to disclose from any of the authors

Funding

The data collection for TTRIS was funded by grants from the Swedish National Board of Health and Welfare, grant numbers 22464/2017 and 23745/2016.

Author Contribution

Authors AA, RD, BS, SD and MGW have conceptualized the study. AA and RD analysed the data and AA, RD, BS were involved in the interpretation of the data and manuscript writing. GR, NR, MK, JA, KDS, NS, MM, AG, NR, and MGW contributed to the study design and critical revisions to the manuscript. All authors have contributed to drafting the article and revising it. They also approved the final version of the manuscript. All authors agree to be responsible for all aspects of the work.

For peer review only - http://bmjopen.bmj.com/site/about/guidelines.xhtml

Acknowledgments

We thank the Thursday Truth Seekers (TTS) and the Trauma Triage Study in India (TTRIS), India, research team for their support.

REFERENCES

1. GBD 2017 Causes of Death Collaborators. Global, regional, and national age-sex-specific mortality for 282 causes of death in 195 countries and territories, 1980-2017: a systematic analysis for the Global Burden of Disease Study 2017. Lancet (London, England) [Internet]. 2018 Nov 10 [cited 2021 Aug 23];392(10159):1736–88. Available from: https://pubmed.ncbi.nlm.nih.gov/30496103/

2. World Health Organization. WHO country cooperation strategy at a glance : India. 2018. Available from: https://apps.who.int/iris/handle/10665/136895

3. Government of India. Ministry of Road Transport & Highways Transport Research Wing. Road Accidents in India 2019. 2019; Available from: https://morth.nic.in/sites/default/files/RA_Uploading.pdf

4. G G. Injuries in India: A national perspective. Burden of Disease in India Equitable Development - Healthy Future New Delhi: National Commission on Macroeconomics and Health, Ministry of Health & Family Welfare, Government of India. 2005;325–347. Available from: http://www.drbrambedkarcollege.ac.in/sites/default/files/Burden of disease in India.pdf#page=343

5. Fitzgerald M, Cameron P, Mackenzie C, Farrow N, Scicluna P, Gocentas R, et al. Trauma resuscitation errors and computer-assisted decision support. Archives of surgery

For peer review only - http://bmjopen.bmj.com/site/about/guidelines.xhtml

(Chicago, Ill : 1960) [Internet]. 2011 Feb [cited 2021 Aug 23];146(2):218–25. Available from: https://pubmed.ncbi.nlm.nih.gov/21339436/

6. Cameron P, Gabbe B, Smith K, Mitra B. Triaging the right patient to the right place in the shortest time. British journal of anaesthesia [Internet]. 2014 [cited 2021 Aug 23];113(2):226–33. Available from: https://pubmed.ncbi.nlm.nih.gov/24961786/

7. Twomey M, Wallis LA, Thompson M lou, Myers JE. The South African Triage Scale (adult version) provides reliable acuity ratings. International emergency nursing [Internet]. 2012 Jul [cited 2021 Aug 23];20(3):142–50. Available from: https://pubmed.ncbi.nlm.nih.gov/22726946/

8. Ebrahimi M, Heydari A, Mazlom R, Mirhaghi A. The reliability of the Australasian Triage Scale: a meta-analysis. World journal of emergency medicine [Internet]. 2015 [cited 2021 Aug 23];6(2):94. Available from: https://pubmed.ncbi.nlm.nih.gov/26056538/

9. Roy N, Murlidhar V, Chowdhury R, Patil SB, Supe PA, Vaishnav PD, et al. Where there are no emergency medical services-prehospital care for the injured in Mumbai, India. Prehospital and disaster medicine [Internet]. 2010 [cited 2021 Aug 23];25(2):145–51. Available from: https://pubmed.ncbi.nlm.nih.gov/20467994/

10. Woolard RH, Borron SW MJM. Emergency Department Design. In: Ciottone's Disaster Medicine. Elsevier. 2016;125–130.

11. Kumar S, Kumar S, Gupta B. Urban health: Needs urgent attention. Indian journal of public health [Internet]. 2018 Jul 1 [cited 2021 Aug 23];62(3):214–7. Available from: https://pubmed.ncbi.nlm.nih.gov/30232971/

For peer review only - http://bmjopen.bmj.com/site/about/guidelines.xhtml

12. Bashar MA, Bhattacharya S, Tripathi S, Sharma N, Singh A. Strengthening primary health care through e-referral system. Journal of family medicine and primary care [Internet]. 2019 [cited 2021 Aug 23];8(4):1511–3. Available from: https://pubmed.ncbi.nlm.nih.gov/31143753/

13. Gidwani S, Jena NN. Perspective article for European Journal of Emergency Medicine: emergency medicine in India. European journal of emergency medicine : official journal of the European Society for Emergency Medicine [Internet]. 2020 [cited 2021 Aug 23];27(5):317–8. Available from: https://pubmed.ncbi.nlm.nih.gov/32852409/

14. McHugh M, Tanabe P, McClelland M, Khare RK. More patients are triaged using the Emergency Severity Index than any other triage acuity system in the United States. Academic emergency medicine : official journal of the Society for Academic Emergency Medicine [Internet]. 2012 Jan [cited 2021 Aug 23];19(1):106–9. Available from: https://pubmed.ncbi.nlm.nih.gov/22211429/

15. Gerdin LW, Khajanchi M, Kumar V, Roy N, Saha ML, Soni KD, et al. Comparison of emergency department trauma triage performance of clinicians and clinical prediction models: a cohort study in India. BMJ open [Internet]. 2020 Feb 18 [cited 2021 Aug 23];10(2). Available from: https://pubmed.ncbi.nlm.nih.gov/32075827/

16. Kumar A, Lakshminarayanan D, Joshi N, Vaid S, Bhoi S, Deorari A. Triaging the triage: reducing waiting time to triage in the emergency department at a tertiary care hospital in New Delhi, India. Emergency medicine journal : EMJ [Internet]. 2019 Sep 1 [cited 2021 Aug 23];36(9):558–63. Available from: https://pubmed.ncbi.nlm.nih.gov/31366625/

For peer review only - http://bmjopen.bmj.com/site/about/guidelines.xhtml

17. Mahajan V, Arora S, Kaur T, Gupta S, Guglani V. Unexpected hospitalisations at a 23-hour observation unit in a paediatric emergency department of northern India. Journal of clinical and diagnostic research : JCDR [Internet]. 2013 Jul 1 [cited 2021 Aug 23];7(7):1418–20. Available from: https://pubmed.ncbi.nlm.nih.gov/23998079/

18. Rominski S, Bell SA, Oduro G, Ampong P, Oteng R, Donkor P. The implementation of the South African Triage Score (SATS) in an urban teaching hospital, Ghana. African journal of emergency medicine : Revue africaine de la medecine d'urgence [Internet]. 2014 [cited 2021 Aug 23];4(2):71–5. Available from: https://pubmed.ncbi.nlm.nih.gov/28344927/

19. Bruijns SR, Wallis LA, Burch VC. A prospective evaluation of the Cape triage score in the emergency department of an urban public hospital in South Africa. Emergency medicine journal : EMJ [Internet]. 2008 Jul [cited 2021 Aug 23];25(7):398–402. Available from: https://pubmed.ncbi.nlm.nih.gov/18573947/

20. World Health Organization. The international statistical classification of diseases and health related problems ICD-10: Tabular list v. 1: Tenth revision. 5th ed. Genève, Switzerland: World Health Organization. 2016;

21. Gardner A, Forson PK, Oduro G, Stewart B, Dike N, Glover P, et al. Diagnostic accuracy of the Kampala Trauma Score using estimated Abbreviated Injury Scale scores and physician opinion. Injury [Internet]. 2017 Jan 1 [cited 2021 Aug 23];48(1):177–83. Available from: https://pubmed.ncbi.nlm.nih.gov/27908493/

For peer review only - http://bmjopen.bmj.com/site/about/guidelines.xhtml

22. Mena JH, Sanchez AI, Rubiano AM, Peitzman AB, Sperry JL, Gutierrez MI, et al. Effect of the modified Glasgow Coma Scale score criteria for mild traumatic brain injury on mortality prediction: comparing classic and modified Glasgow Coma Scale score model scores of 13. The Journal of trauma [Internet]. 2011 Nov [cited 2021 Aug 23];71(5):1185–93. Available from: https://pubmed.ncbi.nlm.nih.gov/22071923/

23. Javali RH, Krishnamoorthy, Patil A, Srinivasarangan M, Suraj, Sriharsha. Comparison of Injury Severity Score, New Injury Severity Score, Revised Trauma Score and Trauma and Injury Severity Score for Mortality Prediction in Elderly Trauma Patients. Indian journal of critical care medicine : peer-reviewed, official publication of Indian Society of Critical Care Medicine [Internet]. 2019 [cited 2021 Aug 23];23(2):73–7. Available from: https://pubmed.ncbi.nlm.nih.gov/31086450/

24. Champion HR, Sacco WJ, Copes WS, Gann DS, Gennarelli TA, Flanagan ME. A revision of the Trauma Score. The Journal of trauma [Internet]. 1989 [cited 2021 Aug 23];29(5):623–9. Available from: https://pubmed.ncbi.nlm.nih.gov/2657085/

25. R Core Team. R: A Language and Environment for Statistical Computing. Vienna, Austria: R Foundation for Statistical Computing. 2020; Available from: https://www.r-project.org/

26. Mehmood A, Razzak JA, Kabir S, Mackenzie EJ, Hyder AA. Development and pilot implementation of a locally developed Trauma Registry: lessons learnt in a low-income country. BMC emergency medicine [Internet]. 2013 Mar 21 [cited 2021 Aug 23];13(1):4. Available from: https://pubmed.ncbi.nlm.nih.gov/23517344/

27. Negussie A, Getie A, Manaye E, Tekle T. Prevalence and outcome of injury in patients visiting the emergency Department of Yirgalem General Hospital, Southern Ethiopia. BMC emergency medicine [Internet]. 2018 May 22 [cited 2021 Aug 23];18(1):14. Available from: https://pubmed.ncbi.nlm.nih.gov/29788913/

28. Sarang B, Bhandarkar P, Raykar N, O'Reilly GM, Soni KD, Wärnberg MG, et al. Associations of On-arrival Vital Signs with 24-hour In-hospital Mortality in Adult Trauma Patients Admitted to Four Public University Hospitals in Urban India: A Prospective Multi-Centre Cohort Study. Injury [Internet]. 2021 May 1 [cited 2021 Aug 23];52(5):1158–63. Available from: https://pubmed.ncbi.nlm.nih.gov/33685640/

29. M G, N R, M K, V K, S D, L F-T, et al. Predicting early mortality in adult trauma patients admitted to three public university hospitals in urban India: a prospective multicentre cohort study. PloS one [Internet]. 2014 [cited 2021 Aug 23];9(9). Available from: https://pubmed.ncbi.nlm.nih.gov/25180494/

Figure 1: Study Flowchart

Figure 2: Percentage distribution of different injury mechanisms among injury types (N = 4135)

Figure 3: Patients' disposition from ED as per retrospective triage using CTS (N=4135)

For peer review only - http://bmjopen.bmj.com/site/about/guidelines.xhtml

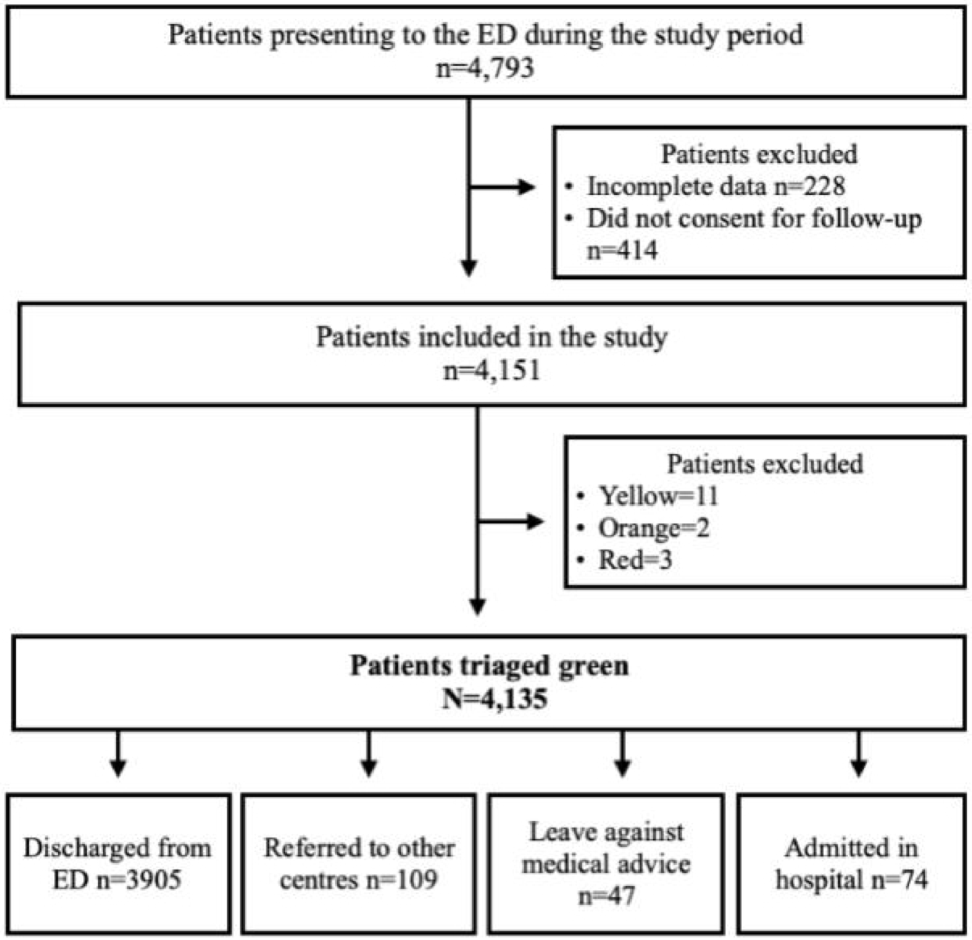

Figure 1: Study Flowchart

52x51mm (300 x 300 DPI)

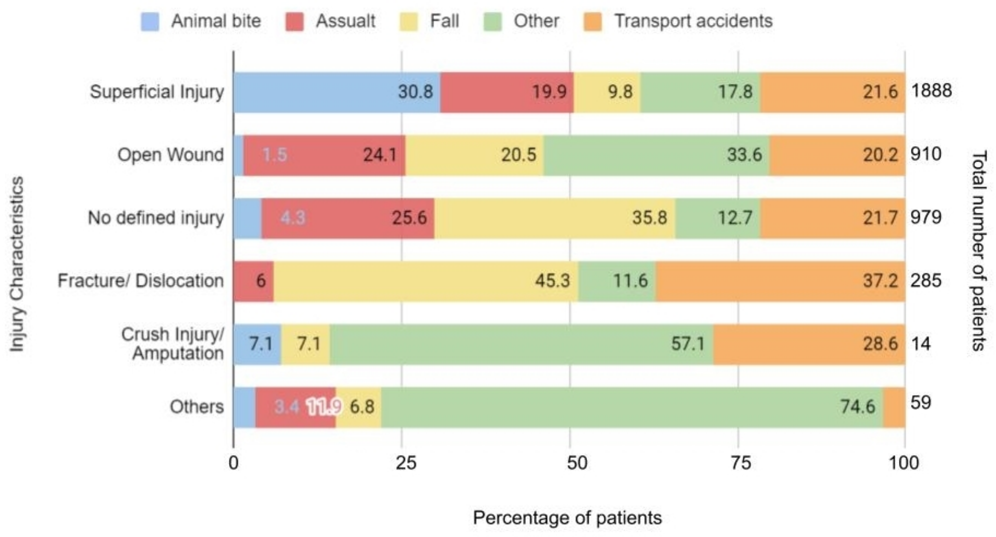

Figure 2: Percentage distribution of different injury mechanisms among injury types (N = 4135)

77x41mm (300 x 300 DPI)

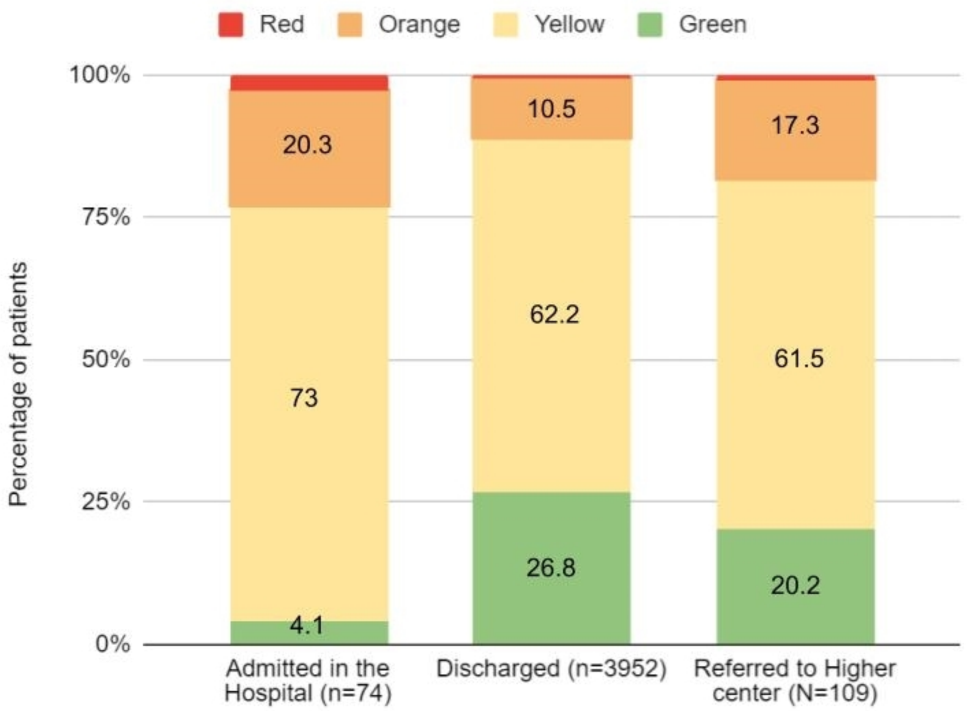

Figure 3: Patients' disposition from ED as per retrospective triage using CTS (N=4135)

61x46mm (300 x 300 DPI)

Supplementary material

Table of excluded International Classification of Diseases version 10 (ICD-10) codes

ICD-10 code	Short description	Full description
W65	Other external causes	Drowning and submersion while in bath-tub
W67	Other external causes	Drowning and submersion while in swimming-pool
W68	Other external causes	Drowning and submersion following fall into swimming-pool
W69	Other external causes	Drowning and submersion while in natural water
W70	Other external causes	Drowning and submersion following fall into natural water
W73	Other external causes	Other specified drowning and submersion
W74	Other external causes	Unspecified drowning and submersion
W78	Other external causes	Inhalation of gastric contents
W79	Other external causes	Inhalation and ingestion of food causing obstruction of respiratory tract
W80	Other external causes	Inhalation and ingestion of other objects causing obstruction of respiratory tract
W81	Other external causes	Confined to or trapped in a low-oxygen environment
W83	Other external causes	Other specified threats to breathing

For peer review only

ICD-10 code	Short description	Full description
W84	Other external causes	Unspecified threat to breathing
X20	Other external causes	Contact with venomous snakes and lizards
X21	Other external causes	Contact with venomous spiders
X22	Other external causes	Contact with scorpions
X23	Other external causes	Contact with hornets, wasps and bees
X24	Other external causes	Contact with centipedes and venomous millipedes (tropical)
X25	Other external causes	Contact with other venomous arthropods
X26	Other external causes	Contact with venomous marine animals and plants
X27	Other external causes	Contact with other specified venomous animals
X28	Other external causes	Contact with other specified venomous plants
X29	Other external causes	Contact with unspecified venomous animal or plant
X40	Other external causes	Accidental poisoning by and exposure to nonopioid analgesics, antipyretics and antirheumatics
X41	Other external causes	Accidental poisoning by and exposure to antiepileptic, sedative-hypnotic, antiparkinsonism and psychotropic drugs, not elsewhere classified
X42	Other external causes	Accidental poisoning by and exposure to narcotics and psychodysleptics [hallucinogens], not elsewhere classified

For peer review only

ICD-10 code	Short description	Full description
X43	Other external causes	Accidental poisoning by and exposure to other drugs acting on the autonomic nervous system
X44	Other external causes	Accidental poisoning by and exposure to other and unspecified drugs, medicaments and biological substances
X45	Other external causes	Accidental poisoning by and exposure to alcohol
X46	Other external causes	Accidental poisoning by and exposure to organic solvents and halogenated hydrocarbons and their vapours
X47	Other external causes	Accidental poisoning by and exposure to other gases and vapours
X48	Other external causes	Accidental poisoning by and exposure to pesticides
X49	Other external causes	Accidental poisoning by and exposure to other and unspecified chemicals and noxious substances
X50	Other external causes	Overexertion and strenuous or repetitive movements
X51	Other external causes	Travel and motion
X52	Other external causes	Prolonged stay in weightless environment
X53	Other external causes	Lack of food
X54	Other external causes	Lack of water
X57	Other external causes	Unspecified privation
X60	Intentional self-harm	Intentional self-poisoning by and exposure to nonopioid analgesics, antipyretics and antirheumatics

For peer review only

X61	Intentional self-harm	Intentional self-poisoning by and exposure to antiepileptic, sedative-hypnotic, antiparkinsonism and psychotropic drugs, not elsewhere classified
X62	Intentional self-harm	Intentional self-poisoning by and exposure to narcotics and psychodysleptics [hallucinogens], not elsewhere classified
X63	Intentional self-harm	Intentional self-poisoning by and exposure to other drugs acting on the autonomic nervous system
X64	Intentional self-harm	Intentional self-poisoning by and exposure to other and unspecified drugs, medicaments and biological substances
X65	Intentional self-harm	Intentional self-poisoning by and exposure to alcohol
X66	Intentional self-harm	Intentional self-poisoning by and exposure to organic solvents and halogenated hydrocarbons and their vapours
ICD-10 code	Short description	Full description
X67	Intentional self-harm	Intentional self-poisoning by and exposure to other gases and vapours
X68	Intentional self-harm	Intentional self-poisoning by and exposure to pesticides
X69	Intentional self-harm	Intentional self-poisoning by and exposure to other and unspecified chemicals and noxious substances
X85	Assault	Assault by drugs, medicaments and biological substances
X88	Assault	Assault by gases and vapours
X89	Assault	Assault by other specified chemicals and noxious substances
X90	Assault	Assault by unspecified chemical or noxious substance
Y06	Assault	Neglect and abandonment
Y060	Assault	Neglect and abandonment by spouse or partner

For peer review only

Y061	Assault	Neglect and abandonment by parent
Y062	Assault	Neglect and abandonment by acquaintance or friend
Y068	Assault	Neglect and abandonment by other specified persons
Y069	Assault	Neglect and abandonment by unspecified person
Y07	Assault	Other maltreatment
Y070	Assault	Other maltreatment by spouse or partner
Y071	Assault	Other maltreatment by parent
Y072	Assault	Other maltreatment by acquaintance or friend
Y073	Assault	Other maltreatment by official authorities
Y078	Assault	Other maltreatment by other specified persons
Y079	Assault	Other maltreatment by unspecified person
Y10	Event of undetermined intent	Poisoning by and exposure to nonopioid analgesics, antipyretics and antirheumatics, undetermined intent
ICD-10 code	Short description	Full description
Y11	Event of undetermined intent	Poisoning by and exposure to antiepileptic, sedative-hypnotic, antiparkinsonism and psychotropic drugs, not elsewhere classified, undetermined intent
Y12	Event of undetermined intent	Poisoning by and exposure to narcotics and psychodysleptics [hallucinogens], not elsewhere classified, undetermined intent
Y13	Event of undetermined intent	Poisoning by and exposure to other drugs acting on the autonomic nervous system, undetermined intent
Y14	Event of undetermined intent	Poisoning by and exposure to other and unspecified drugs, medicaments and biological substances, undetermined intent

For peer review only

Y15	Event of undetermined intent	Poisoning by and exposure to alcohol, undetermined intent
Y16	Event of undetermined intent	Poisoning by and exposure to organic solvents and halogenated hydrocarbons and their vapours, undetermined intent
Y17	Event of undetermined intent	Poisoning by and exposure to other gases and vapours, undetermined intent
Y18	Event of undetermined intent	Poisoning by and exposure to pesticides, undetermined intent
Y19	Event of undetermined intent	Poisoning by and exposure to other and unspecified chemicals and noxious substances, undetermined intent
Y352	Legal intervention and operations of war	Legal intervention involving gas
Y355	Legal intervention and operations of war	Legal execution

For peer review only

STROBE 2007 (v4) Statement—Checklist of items that should be included in reports of *cohort studies*

Section/Topic	Item #	Recommendation	Reported on page #
**Title and abstract**	1	(*a*) Indicate the study's design with a commonly used term in the title or the abstract	1
		(*b*) Provide in the abstract an informative and balanced summary of what was done and what was found	3
**Introduction**			
Background/rationale	2	Explain the scientific background and rationale for the investigation being reported	5
Objectives	3	State specific objectives, including any prespecified hypotheses	6
**Methods**			
Study design	4	Present key elements of study design early in the paper	6
Setting	5	Describe the setting, locations, and relevant dates, including periods of recruitment, exposure, follow-up, and data collection	6
Participants	6	(*a*) Give the eligibility criteria, and the sources and methods of selection of participants. Describe methods of follow-up	8
		(*b*) For matched studies, give matching criteria and number of exposed and unexposed	-
Variables	7	Clearly define all outcomes, exposures, predictors, potential confounders, and effect modifiers. Give diagnostic criteria, if applicable	9 - 10
Data sources/ measurement	8*	For each variable of interest, give sources of data and details of methods of assessment (measurement). Describe comparability of assessment methods if there is more than one group	8
Bias	9	Describe any efforts to address potential sources of bias	-
Study size	10	Explain how the study size was arrived at	-
Quantitative variables	11	Explain how quantitative variables were handled in the analyses. If applicable, describe which groupings were chosen and why	12-13
Statistical methods	12	(*a*) Describe all statistical methods, including those used to control for confounding	12-13
		(*b*) Describe any methods used to examine subgroups and interactions	-
		(*c*) Explain how missing data were addressed	12
		(*d*) If applicable, explain how loss to follow-up was addressed	-
		(*e*) Describe any sensitivity analyses	-
**Results**			

For peer review only

Participants	13*	(a) Report numbers of individuals at each stage of study—eg numbers potentially eligible, examined for eligibility, confirmed eligible, included in the study, completing follow-up, and analysed	13
		(b) Give reasons for non-participation at each stage	-
		(c) Consider use of a flow diagram	Figure 1
Descriptive data	14*	(a) Give characteristics of study participants (eg demographic, clinical, social) and information on exposures and potential confounders	13
		(b) Indicate number of participants with missing data for each variable of interest	-
		(c) Summarise follow-up time (eg, average and total amount)	-
Outcome data	15*	Report numbers of outcome events or summary measures over time	15 -16
Main results	16	(*a*) Give unadjusted estimates and, if applicable, confounder-adjusted estimates and their precision (eg, 95% confidence interval). Make clear which confounders were adjusted for and why they were included	-
		(*b*) Report category boundaries when continuous variables were categorized	-
		(*c*) If relevant, consider translating estimates of relative risk into absolute risk for a meaningful time period	-
Other analyses	17	Report other analyses done—eg analyses of subgroups and interactions, and sensitivity analyses	16-17
**Discussion**			
Key results	18	Summarise key results with reference to study objectives	17-20
**Limitations**			
Interpretation	20	Give a cautious overall interpretation of results considering objectives, limitations, multiplicity of analyses, results from similar studies, and other relevant evidence	20
Generalisability	21	Discuss the generalisability (external validity) of the study results	20
**Other information**			
Funding	22	Give the source of funding and the role of the funders for the present study and, if applicable, for the original study on which the present article is based	22

*Give information separately for cases and controls in case-control studies and, if applicable, for exposed and unexposed groups in cohort and cross-sectional studies.

Note: An Explanation and Elaboration article discusses each checklist item and gives methodological background and published examples of transparent reporting. The STROBE checklist is best used in conjunction with this article (freely available on the Web sites of PLoS Medicine at http://www.plosmedicine.org/, Annals of Internal Medicine at http://www.annals.org/, and Epidemiology at http://www.epidem.com/). Information on the STROBE Initiative is available at www.strobe-statement.org.

For peer review only

BMJ Open

Profile and Triage Appropriateness of Trauma Patients Triaged Green: A Prospective Cohort Study from a Secondary Care Hospital in India

Journal:	*BMJ Open*
Manuscript ID	bmjopen-2022-065036.R1
Article Type:	Original research
Date Submitted by the Author:	10-Oct-2022
Complete List of Authors:	Anthony, Aroke; Doctors For You<br>Dutta, Rohini; - World Health Organization Collaborating Center for Research in Surgical Care Delivery in Low-and-Middle Income Countries; - Christian Medical College and Hospital,<br>Sarang, Bhakti; World Health Organization Collaborating Centre on Research in Surgical Care Delivery in Low-Middle-Income Countries; Terna Medical College & Hospital, Department of Surgery<br>David, Siddarth; Karolinska Institutet, Department of Global Public Health<br>O'Reilly, Gerard; Monash University, Emergency medicine<br>Raykar, Nakul; Harvard Medical School, Department of Global Health and Social Medicine; Brigham and Women's Hospital, Emergency Surgery<br>Khajanchi, Monty; Seth Gowardhandas Sunderdas Medical College and King Edward Memorial Hospital, Department of Surgery<br>Attergrim, Jonatan; Karolinska Institutet<br>Soni, Kapil; All India Institute of Medical Sciences, Critical Care<br>Sharma, Naveen; All India Institute of Medical Sciences, Surgery<br>Mohan, Monali; WHO Collaboration Centre for Research in Surgical Care Delivery in LMICs, Mumbai, India,<br>Gadgil, Anita; World Health Organization Collaborating Center for Research in Surgical Care Delivery in Low-and-Middle Income Countries<br>Roy, Nobhojit; World Health Organization Collaborating Center for Research in Surgical Care Delivery in Low-and-Middle Income Countries<br>Gerdin Wärnberg, Martin ; Karolinska Institutet, Department of Global Public Health; Karolinska University Hospital
**Primary Subject Heading**:	Emergency medicine
Secondary Subject Heading:	Emergency medicine, Public health
Keywords:	TRAUMA MANAGEMENT, ACCIDENT & EMERGENCY MEDICINE, Trauma management < ORTHOPAEDIC & TRAUMA SURGERY

SCHOLARONE™
Manuscripts

I, the Submitting Author has the right to grant and does grant on behalf of all authors of the Work (as defined in the below author licence), an exclusive licence and/or a non-exclusive licence for contributions from authors who are: i) UK Crown employees; ii) where BMJ has agreed a CC-BY licence shall apply, and/or iii) in accordance with the terms applicable for US Federal Government officers or employees acting as part of their official duties; on a worldwide, perpetual, irrevocable, royalty-free basis to BMJ Publishing Group Ltd ("BMJ") its licensees and where the relevant Journal is co-owned by BMJ to the co-owners of the Journal, to publish the Work in this journal and any other BMJ products and to exploit all rights, as set out in our licence.

The Submitting Author accepts and understands that any supply made under these terms is made by BMJ to the Submitting Author unless you are acting as an employee on behalf of your employer or a postgraduate student of an affiliated institution which is paying any applicable article publishing charge ("APC") for Open Access articles. Where the Submitting Author wishes to make the Work available on an Open Access basis (and intends to pay the relevant APC), the terms of reuse of such Open Access shall be governed by a Creative Commons licence – details of these licences and which Creative Commons licence will apply to this Work are set out in our licence referred to above.

Other than as permitted in any relevant BMJ Author's Self Archiving Policies, I confirm this Work has not been accepted for publication elsewhere, is not being considered for publication elsewhere and does not duplicate material already published. I confirm all authors consent to publication of this Work and authorise the granting of this licence.

For peer review only

Profile and Triage Appropriateness of Trauma Patients Triaged Green: A Prospective Cohort Study from a Secondary Care Hospital in India

Aroke Anna Anthony,[1,2] Rohini Dutta,[2,3] Bhakti Sarang,[2,4] Siddarth David,[5] Gerard O'Reilly,[6,7,8] Nakul Raykar,[9,10,11] Monty Khajanchi,[2,12] Jonatan Attergrim,[13] Kapil Dev Soni,[14] Naveen Sharma,[15] Monali Mohan,[2] Anita Gadgil,[2] Nobhojit Roy,[2,5,7] Martin Gerdin Wärnberg[5,13]

[1] Doctors For You, Mumbai, India

[2] Trauma Research Group, World Health Organization Collaborating Centre on Research in Surgical Care Delivery in Low-Middle-Income Countries, Mumbai, India

[3] MBBS, Christian Medical College and Hospital, Ludhiana, India

[4] Department of Surgery, Terna Medical College & Hospital, New Mumbai, India

[5] Department of Global Public Health, Karolinska Institutet, Stockholm, Sweden

[6] Epidemiology & Biostatistics, National Trauma Research Institute, The Alfred, Melbourne, Australia

[7] School of Public Health & Preventive Medicine, Monash University, Melbourne, Australia

[8] Global programs, Emergency & Trauma Centre, The Alfred, Melbourne, Australia

[9] Program in Global Surgery and Social Change, Harvard Medical School, Boston, USA

[10] Trauma, Emergency Surgery & Surgical Critical Care, Brigham and Women's Hospital, Boston, MA, USA

[11] Center for Surgery and Public Health, Brigham and Women's Hospital, Boston, MA, USA

[12] Department of Surgery, Seth.G.S. Medical College & K.E.M. Hospital, Parel, Mumbai, India

[13] Function Perioperative Medicine and Intensive Care, Karolinska University Hospital, Solna, Sweden

[14] Critical & Intensive Care, JPN Apex Trauma Centre, AIIMS, New Delhi, India

[15] Department of Surgery, All India Institute of Medical Sciences, Jodhpur, India

Corresponding Author: Martin Gerdin Wärnberg

Department of Global Public Health, Karolinska Institutet, Stockholm, Sweden; Function Perioperative Medicine and Intensive Care, Karolinska University Hospital, Solna, Sweden.

Address: Department of Global Public Health
Karolinska Institutet
171 77 Stockholm
Email: martin.gerdin@ki.se
Phone Number: +46 708539598

Short title: Profile and Triage Appropriateness of Trauma Patients Triaged Green in a Secondary Care Hospital in India

Word Count (Abstract): 262 words

For peer review only - http://bmjopen.bmj.com/site/about/guidelines.xhtml

Word Count (Manuscript): 3086 words

For peer review only

For peer review only - http://bmjopen.bmj.com/site/about/guidelines.xhtml

ABSTRACT

Objective: To evaluate the profile of non-urgent patients triaged 'green', as part of a triage-trial in the Emergency Department (ED) of a secondary-care hospital in India. The secondary aim was to validate our findings with the Cape Triage Score (CTS).

Design: Prospective cohort study

Setting: A secondary care-hospital in Mumbai, India.

Participants: Patients aged 18 years and above with a history of trauma defined as having any of the external causes of morbidity and mortality listed in block V01-Y36, chapter XX of the International Classification of Disease version 10 (ICD-10) codebook, triaged green between July 2016 to November 2019.

Outcome: Outcome measures were mortality within 24-hours, 30-days and mistriage.

Results: We included 4135 trauma patients triaged green. The mean age of patients was 32.8 (±13.1) years, and 77% were males. The median (IQR) length of stay of admitted patients was 3 (13) days. Half the patients had a mild Injury Severity Score (3-8), with the majority of injuries being blunt (98%). Of the patients triaged green by clinicians, three-quarters (74%) were undertriaged on cross validating with CTS. On telephonic follow-up two patients were reported dead whereas one died while admitted in-hospital.

Conclusion: Our study highlights the need for implementation and evaluation of training in trauma triage systems that use physiological parameters including pulse, systolic blood pressure and glascow coma scale, for the in-hospital first responders (clinicians, nurses and other paramedical staff) in the EDs.

Ethical clearance: Ethics committee approval for Trauma Triage Study in India (TTRIS) was obtained from the ethics and scientific committee of Khurshedji Behramji Bhabha Hospital (KBBH) (KBBH, HO/4982/KBB,12/08/2016).

For peer review only - http://bmjopen.bmj.com/site/about/guidelines.xhtml

Strengths and limitations of this study:

- This is a prospective cohort study, with vital signs recorded by a dedicated research officer, documenting the profile of green triaged patients from a public secondary care hospital in an urban LMIC setting conducted over a period of 3 years.
- Triage appropriateness was assessed using a standardised and validated triage scoring system (CTS), that included both physiological parameters and injury characteristics of the patients.
- The study provides data from a single secondary care hospital in Mumbai. Therefore, the results cannot be generalised to other Indian hospitals due to hospital bias.
- Data of only the first 10 consecutive patients were collected each shift during the study period.
- We lack data documenting 30-day mortality of all the patients and morbidity.

For peer review only

For peer review only - http://bmjopen.bmj.com/site/about/guidelines.xhtml

INTRODUCTION

Globally, each year, 4.5 million people die from trauma, with India contributing to 20% of this burden. [1] Trauma represents the second most common cause of death after age five in India.[2] Furthermore, India also has the highest rank in the number of deaths attributable to road traffic-related deaths in the world.[3] Approximately 50% of trauma deaths occur in the hospitals, highlighting the need for strengthening in-hospital care.[4] Trauma care is highly time-sensitive, and the early identification of injuries is important for survival.[5] Establishment of hospital triage systems can ensure that critically ill patients are identified and receive care promptly.[6] For this purpose, several triage scores are used across different countries and hospital settings.[7,8]

In India, the high population density, poorly developed prehospital care and a lack of appropriate referral systems leads to overcrowding in the emergency departments (EDs).[9–12] Most EDs lack triage protocols and the level of emergency patient care is decided by clinicians who are not trained specifically in trauma care.[13,14] Lack of adequate triage contributes to a surge in non-urgent patients presenting directly to the ED, exacerbating the problem of overcrowding.[14–17] This leads to diversion of workforce and resources from serious patients requiring immediate care. Nonetheless, not evaluating these patients could result in missed injuries and poor outcomes.

In our study, clinicians at a triage-naive ED were introduced to a triage-trial, as part of a multicentre triage project which compared prediction models for triage in adult trauma patients presenting to various emergency departments across India.[15] The patients were designated one of the four trauma triage categories by clinicians, based on their understanding of trauma triage; into red, orange, yellow, green, with red and green denoting the most and least urgent patient status respectively. We aimed to evaluate the profile of the non-urgent patients who were triaged green by clinicians and the validity of this category in comparison to the Cape Triage Score (CTS). CTS is a mixed score based on physiological parameters and the pathology of the patient and has been effectively used at various settings in South Africa and Low- and

For peer review only

Middle-Income Countries (LMIC) since 2006.[18] It is a comprehensive triage score with a low undertriage rate capable of predicting patient disposition.[19]

METHODOLOGY

Study Design

This single-centre prospective cohort study is part of the Trauma Triage Study in India (TTRIS) which compares prediction models for triage in adult trauma patients presenting to various emergency departments across India. Data was collected in the study site from July 2016 to November 2019.

Study Setting

The study site was the ED of Khurshedji Behramji Bhabha Hospital (KBBH), a 436 bedded regional secondary healthcare centre located in Mumbai, India, catering to approximately 350 patients each day in the ED. It is a secondary-care public hospital with free or nominal fees, providing access to low socio-economic groups and receives patients from across the city. At KBBH, trauma care is imparted as a subspeciality along with medical, surgical, and obstetric care. The hospital has an intensive care facility but there is no neurosurgery department, so patients in need of neurosurgical management are referred to tertiary care centres. Plain radiography and ultrasonography are available round the clock; however computerised tomography (CT) is only available in-house from 7am to 6pm. The patients arriving at the ED are first seen by a casualty medical officer (CMO) largely on a first-come, first-served basis without a formalised system of triaging patients at the ED.

Clinician Triage

As part of data collection of TTRIS, the triage-naive clinicians were informed about the trauma triage categories, without provision to any formal tool or training about the same. The clinicians involved have a minimum of 2 years clinical experience, however, they are neither trained in trauma care as a speciality nor are they necessarily trained in trauma management courses like Advanced Trauma Life Support (ATLS). Medical training of the clinicians includes aspects such as triage systems in theory however not

For peer review only - http://bmjopen.bmj.com/site/about/guidelines.xhtml

put in practise due to lack of formal triage protocols. After their initial on-arrival assessment of each patient, the research officers asked the clinicians to categorise the urgency of patients into the aforementioned colour-coded triage groups,[15] henceforth referred to as the triage levels. The triage level denoted by clinicians was just based on their experiential and intuitive clinical knowledge. For doing this, the clinicians were allowed to use all available information that was extracted by them during initial routine assessment. The triage levels were not used to determine treatment decisions in the ED as there was no formalised tool or protocol in place for assigning the triage and coupling it with patient management. No formal training in triage was given except for standard comparable labels to different triage colour categories, in order to allow them to continue with their routine intuitive clinical assessment. The clinicians were individually informed about the aim and methodology used for the TTRIS study at the start of their respective posting at the ED, however, the clinicians were neither involved in the conception nor were they part of the research team analysing the results.

Participants

Inclusion Criteria: We included all the patients aged 18 years and above presenting to the KBBH ED with a history of trauma as their primary complaint and triaged green by clinical triage on initial evaluation irrespective of their injury severity. A history of trauma was defined here as having any of the external causes of morbidity and mortality listed in block V01-Y36, chapter XX of the International Classification of Disease version 10 (ICD-10) online codebook, with some exclusions (see online supplementary material).[20]

Exclusion Criteria: Patients with missing data in one or more variables used for analysis or who did not consent to follow-up were excluded from the analysis.

Source and methods of selection of participants and follow-up

The research officer at KBBH observed morning, evening and night shifts (6-hour observational shifts). These shifts were not aligned with the working hours of the clinical staff to reduce bias and accounting for shift fatigue of the clinicians. Data were collected from the first 10 consecutive patients only, irrespective

For peer review only

of their triage, during each shift. The research officer collected the vital signs but was in no way involved in patient assessment or management. Due to the large patient load and time and budgetary constraints of the project, data collection of only the first 10 patients was considered feasible for follow-up.

The research officer performed follow-up at 24-hours, 30-days after arrival at the ED. The follow-up was completed in-person or by telephone, depending on whether the patient was still hospitalised or if the patient had been discharged. The phone numbers of one or more contact persons, mostly relatives, were collected on enrolment and those people were contacted if the participant did not reply to the follow-up telephone calls. The outcome was recorded as missing if neither the patient nor the relative were available for follow-up at the specified time-points.

Variables

To evaluate the profile of patients triaged green we analysed the 24 hours and 30 days mortality. To determine the triage appropriateness, we retrospectively used CTS. Additionally, for each participant, age, sex, mechanism of injury, injury-related details, assigned informal triage category, and intensive care unit (ICU) or ward admission status were collected. Physiological measures including systolic blood pressure (SBP), respiratory rate (RR), heart rate (HR), peripheral capillary oxygen saturation (SpO_2), Glasgow Coma Scale (GCS) and Alert Verbal Pain unresponsive scale (AVPU) were recorded.

GCS was categorised into no or mild traumatic brain injury (TBI) (13-15), moderate TBI (9-12), severe TBI (3-8).[21] Length of stay in the hospital (LoS) was calculated using the data and time of admission in the hospital to the data and time of discharge alive from the hospital, mortality, leave against medical advice (LAMA) or abscond. Injury severity score (ISS) was allocated retrospectively with 'mild' (3-8), 'moderate' (9-15), 'severe' (16-25) and 'profound' (>25) categories. Patients for whom ISS could not be coded, for example when there were no recorded injuries, were assigned 'no defined ISS'.[22] The revised trauma score (RTS) was computed and categorised as RTS < 4 and RTS > 4.[22,23]

Injuries were recorded and coded using ICD-10 in the TTRIS dataset. Patients were divided into categories with respect to the most critical injury namely, crush injury, injury to internal organs, blood vessel injury,

For peer review only - http://bmjopen.bmj.com/site/about/guidelines.xhtml

amputation, fracture, dislocation, burn, multiple injury, unspecified injury, open wound, superficial injury.[20] Injury characteristics of patients that presented to the ED with no injuries were categorized as 'no defined injury'. For patients with multiple injuries, the more critical one was considered for categorising patients as per injury. Time of arrival of patients was categorised into four groups, namely, morning (6am-11:59am), afternoon (12pm – 5:59pm), evening (6pm – 11:59pm), and night (12am – 5:59 am).[24] The adult CTS was used retrospectively to check for the appropriateness of the informal triage performed by the clinicians.[18] The patients triaged green as per the clinician triage were further classified using the Triage Early Warning Score (TEWS) and South African Triage Score (SATS) colour code into red, orange, yellow and green.

Quality assurance

There were three layers of quality control. First, data was entered using a dedicated electronic data collection instrument with extensive logical checks and prompts for unlikely but possible values. Second, the collected data were reviewed on a weekly basis and discussed during weekly online conferences with all research officers and the project leads throughout the duration of the data collection period. Third, on-site quality control sessions were conducted every 3-4 months. During these sessions, a second research officer collected data alongside the research officer who worked at the ED. The quality-controlled data was then compared with the standard data.

Patient and Public Involvement

No patients were involved.

Data Analysis

Data analysis was performed using R version 4.04 statistical software.[25] Complete case analysis was performed to include patients with complete data. We describe the sample using frequencies and percentages for categorical variables, and mean and standard deviation (SD) for normally distributed continuous variables and median and inter quartile range (IQR) for non-normally distributed continuous variables. The number of patients triaged green by the CTS was divided by the number of patients triaged

For peer review only - http://bmjopen.bmj.com/site/about/guidelines.xhtml

green as per clinician triage (4135), the resultant proportion minus one was considered as the proportion of patients mis-triaged.

RESULTS

In the study, 4151 patients were included of which 4135 (99.6%) patients were triaged green by the clinicians **(Figure 1)**.

Profile of patients triaged green

The mean age of patients was 32.8±13.1 years with 3172 (77%) males. Notably, of all patients triaged green, 10/4135 (0.24%) patients presented with moderate to severe GCS and 0.3% of patients did not have an AVPU of alert. Majority of patients (97%) triaged green presented to the study-site directly without a primary care hospital referral.

Of the total patients triaged green by clinicians, 46% of patients had only superficial injuries of which majority (30.8%) were due to animal bites. Further, 24% had no external injuries on examination. Among those referred to other centers, the most common types of injury identified were superficial injuries (34) followed by open wounds (27) and patients with no documented injury (19). The reasons for referral to other centres were not documented. As per ISS, 50.2% of patients had 'mild' and 0.4% had 'moderate' score and the remaining 49.5% patients had 'no defined ISS'. **Figure 2** shows the different injury types as per mechanism of injury in the study population. Amongst those that had a transport accident, 881/916 (96.17%) were patients who had a road traffic injury.

The ED disposition of all the patients is shown in **Figure 1**. The median (IQR) length of stay (LoS) of those admitted to the hospital was 3 (13) days and seven patients required admission in the ICU. Most admitted patients 62/74 (83.8%) were successfully discharged from the hospital while three were transferred to other centers for further management. Further, there were eight patients that left against medical advice and one who died during their hospital stay.

For peer review only - http://bmjopen.bmj.com/site/about/guidelines.xhtml

Follow up at 30 days was successful for 3832/4135 (92.7%) of patients. Three patients died during the first 30 days. Of these patients, two had a GCS of <8 on initial evaluation. The CTS triage of these patients were yellow and orange as per the CTS.

Evaluation of triage appropriateness through retrospective CTS

We found that of the total number of patients that were triaged green by clinicians (N=4135), 24 patients were triaged red, 448 patients were triaged orange and 2579 patients were triaged yellow as per CTS indicating that 73.8% patients were mistriaged by the ED clinicians. Proportions of mistriage were higher during the night and afternoon (**Table 1A**). Of these, most patients (97%) were found to have been mistriaged after assessing their physiological parameters from TEWS while others due to the SATS color code for discriminators as seen in **Table 1B**. In **Figure 3** the disposition of these patients from the ED as per their CTS is depicted. Notably, of the total three documented deaths, one occurred in a patient who was admitted in the hospital and triaged orange as per CTS, and one in a patient transferred to a different centre triaged yellow as per CTS. **Table 1C** shows that, CTS was successfully able to triage patients with fractures, dislocation and amputations as urgent.

Table 1 A: Patients mistriaged as per the time of the day (reference)

Mistriage	Morning	Afternoon	Evening	Night	p
n	756	1576	1155	648	
**True**	511 (67.6)	1185 (75.2)	858 (74.3)	497 (76.7)	<0.001
**False**	245 (32.4)	391 (24.8)	297 (25.7)	151 (23.3)	

Table 1 B: Patients mistriaged as per CTS (N=4135)

Cape Triage Score	Green	Yellow	Orange	Red
n	1084	2579	448	24
**Triage Early Warning Score**	1084	2513	433	19

For peer review only - http://bmjopen.bmj.com/site/about/guidelines.xhtml

**South African Triage Scale**	0	66	15	5

Table 1 C: Injury characteristics as per the CTS Triage category

	**CTS Triage**	**Green**	**Yellow**	**Orange**	**Red**	**p**
**Injury Type**	Amputation/ Crush Injury	0 (0.0)	13 (0.5)	1 (0.2)	0 (0.0)	<0.001
	Fracture/Dislocation	0 (0.0)	243 (9.4)	41 (9.2)	1 (4.2)	
	Others	9 (0.8)	36 (1.4)	9 (2.0)	5 (20.8)	
	Open wound	204 (18.8)	575 (22.3)	123 (27.5)	8 (33.3)	
	Superficial Injury	635 (58.6)	1074 (41.6)	174 (38.8)	5 (20.8)	
	No defined Injury	236 (21.8)	638 (24.7)	100 (22.3)	5 (20.8)	

For peer review only

DISCUSSION

Blunt trauma was seen as the most common type of injury.[26] Transport accidents was the predominant mechanism of injury and 77% patients were males. Most patients had mild ISS (50.2%) and only about 0.4% patients had moderate ISS with no patients in the severe and profound ISS category. Most patients presented with seemingly superficial injuries. The presentation of 15.5% of patients with animal bites was unique to our setting. These patients mainly presented for vaccinations following animal bites more frequently than for the treatment of bite injuries.

Our study documented that approximately three quarters (74%) of patients informally triaged green were effectively mistriaged when compared to CTS. Out of 4135 patients triaged green by clinicians only 1084/4135 (26.2%) were triaged green according to the CTS. Most of these patients (97%) were coming in as direct arrival to this secondary-care hospital. CTS also rightly identified patients with fractures and dislocation as urgent thus avoiding the possibility of having missed injuries. These factors emphasise the need of on-scene triage and an effective referral system.

For peer review only - http://bmjopen.bmj.com/site/about/guidelines.xhtml

Our study found that 10/4135 (0.24%) patients that reported GCS moderate to severe and 0.3% of patients did not have an AVPU of alert and were still triaged green. This indicates that among the green triaged patients with close to normal physiological parameters, there were patients that required urgent attention. Although the proportion of these patients is relatively low compared to our sample size, reasons for these patients being inappropriately triaged must be explored extensively to enhance healthcare delivery in Indian EDs. Additionally, these findings highlight the efficacy of physiological scores such as TEWS, a component of CTS in triaging patients accurately and the need to include GCS assessment for all patients presenting to the ED. Although the reasons for mistriaging are multifactorial, in this case, the lack of appropriate training or standard, uniform protocol for patient management in the ED to quickly identify these patients among those that have normal physiological parameters is most evident. The other factor, overcrowding of the ED with limitation of resources, may also lead to inadequate trauma care.[27] This makes triaging crucial which enables intensifying the efforts towards patients requiring immediate interventions and quick management and disposition of the less urgent patients.

In addition to the high proportion of mistriage ascertained by TEWS, of those admitted, seven required admissions to the ICU indicating they may have required urgent management for their condition. On a closer look at the triage category denoted by clinicians at the three patients found dead on 30-day follow-up, it was seen that two of them were under triaged on initial evaluation of physiological parameters as they had a GCS < 8. Including appropriate triage training for CMO's when implementing reforms in trauma management is a key step towards improving outcomes in trauma patients. This requires prioritisation of meticulous evaluation of the initial vital parameters by the ED staff to reduce errors and improve outcomes, in addition to addressing other contributing factors such as the low clinician to patient ratio in Indian EDs.

This is a prospective cohort study, with vital signs recorded by a dedicated research officer, documenting the profile of green triaged patients from a public secondary care hospital in an urban LMIC setting conducted over a period of 3 years. Triage appropriateness was assessed using a standardised and validated triage scoring system (CTS), that included both physiological parameters and injury characteristics of the

For peer review only - http://bmjopen.bmj.com/site/about/guidelines.xhtml

patients. This study has a few limitations. Firstly, the study provides data from a single secondary care centre, results of which may not be generalisable to other secondary care hospitals or other Indian healthcare settings. Secondly, to ensure feasibility, data of only 10 consecutive patients were collected in each shift. Lastly, we lack data documenting the 30-day mortality of all patients and have none on morbidity. This is a limiting factor towards assessing the morbidity gains.

CONCLUSION

Three-fourths (74%) of the patients triaged green by clinicians in a secondary care hospital in Mumbai were mistriaged when retrospectively analysed using CTS. This highlights the need for implementation and evaluation of trauma triage training, involving systems that rely on presenting physiological parameters, for clinicians, nurses and other paramedical staff in the EDs. Also, direct admissions of the non-urgent patients to this secondary-care hospital warrants strengthening the referral systems to avoid overcrowding of the Indian EDs the in-hospital first responders.

LIST OF ABBREVIATIONS

Emergency department (ED), Cape Triage Score (CTS), Low- and Middle-Income Country (LMIC), Trauma Triage Study in India (TTRIS), Khurshedji Behramji Bhabha Hospital (KBBH), Computerized Tomography (CT), Casualty Medical Officer (CMO), International Classification of Disease version 10 (ICD-10), Injury Severity Score (ISS), systolic blood pressure (SBP), respiratory rate (RR), heart rate (HR), peripheral capillary oxygen saturation (SpO2), Glasgow Coma Scale (GCS), Alert Verbal Pain unresponsive scaled (AVPU), Traumatic Brain Injury (TBI), ED-length of stay (ED-LoS), Length of stay in the hospital (LoS), Leave against medical advice (LAMA), Revised Trauma Score (RTS), Triage Early Warning Score (TEWS), South African Triage Scale (SATS), Standard deviation (SD), Inter quartile range (IQR)

For peer review only - http://bmjopen.bmj.com/site/about/guidelines.xhtml

DECLARATIONS

Ethical clearance

Ethics committee approval for TTRIS was obtained from the ethics and scientific committee of KBBH (KBBH, HO/4982/KBB,12/08/2016). Informed consent for follow-up was taken from patients at the time of discharge from the hospital. In case the patient was unconscious, consent was obtained from a family member or the patient's legally acceptable representative.

Consent for publication

Not Applicable

Availability of data and materials

The data are available to whoever wants them by emailing the corresponding author. They can write their aims and objectives, and then, the authors can decide if that study can be done without duplication of the work.

Competing Interests

There is no conflict of interest to disclose from any of the authors

Funding

The data collection for TTRIS was funded by grants from the Swedish National Board of Health and Welfare, grant numbers 22464/2017 and 23745/2016.

Author Contribution

Authors AA, RD, BS, SD and MGW have conceptualized the study. AA and RD analysed the data and AA, RD, BS were involved in the interpretation of the data and manuscript writing. GR, NR, MK, JA, KDS, NS, MM, AG, NR, and MGW contributed to the study design and critical revisions to the manuscript. All authors have contributed to drafting the article and revising it. They also approved the final version of the manuscript. All authors agree to be responsible for all aspects of the work.

Acknowledgments

For peer review only - http://bmjopen.bmj.com/site/about/guidelines.xhtml

We thank the Thursday Truth Seekers (TTS) and the Trauma Triage Study in India (TTRIS), India, research team for their support.

REFERENCES

1. GBD 2017 Causes of Death Collaborators. Global, regional, and national age-sex-specific mortality for 282 causes of death in 195 countries and territories, 1980-2017: a systematic analysis for the Global Burden of Disease Study 2017. Lancet [Internet]. 2018 Nov 10 [cited 2021 Aug 23];392(10159):1736–88. Available from: https://pubmed.ncbi.nlm.nih.gov/30496103/

2. World Health Organization. WHO country cooperation strategy at a glance : India. 2018. Available from: https://apps.who.int/iris/handle/10665/136895

3. Government of India. Ministry of Road Transport & Highways Transport Research Wing. Road Accidents in India 2019. 2019; Available from: https://morth.nic.in/sites/default/files/RA_Uploading.pdf

4. G G. Injuries in India: A national perspective. Burden of Disease in India Equitable Development - Healthy Future New Delhi: National Commission on Macroeconomics and Health, Ministry of Health & Family Welfare, Government of India. 2005;325–347. Available from: http://www.drbrambedkarcollege.ac.in/sites/default/files/Burden of disease in India.pdf#page=343

5. Fitzgerald M, Cameron P, Mackenzie C, Farrow N, Scicluna P, Gocentas R, et al. Trauma resuscitation errors and computer-assisted decision support. Arch Surg [Internet]. 2011 Feb [cited 2021 Aug 23];146(2):218–25. Available from: https://pubmed.ncbi.nlm.nih.gov/21339436/

6. Cameron P, Gabbe B, Smith K, Mitra B. Triaging the right patient to the right place in the shortest time. Br J Anaesth [Internet]. 2014 [cited 2021 Aug 23];113(2):226–33. Available from: https://pubmed.ncbi.nlm.nih.gov/24961786/

7. Twomey M, Wallis LA, Thompson M lou, Myers JE. The South African Triage Scale (adult version) provides reliable acuity ratings. Int Emerg Nurs [Internet]. 2012 Jul [cited 2021 Aug 23];20(3):142–50. Available from: https://pubmed.ncbi.nlm.nih.gov/22726946/

8. Ebrahimi M, Heydari A, Mazlom R, Mirhaghi A. The reliability of the Australasian Triage Scale: a meta-analysis. World J Emerg Med [Internet]. 2015 [cited 2021 Aug 23];6(2):94. Available from: https://pubmed.ncbi.nlm.nih.gov/26056538/

9. Roy N, Murlidhar V, Chowdhury R, Patil SB, Supe PA, Vaishnav PD, et al. Where there are no emergency medical services-prehospital care for the injured in Mumbai, India. Prehosp Disaster Med [Internet]. 2010 [cited 2021 Aug 23];25(2):145–51. Available from: https://pubmed.ncbi.nlm.nih.gov/20467994/

10. Woolard RH, Borron SW MJM. Emergency Department Design. In: Ciottone's Disaster Medicine. Elsevier. 2016;125–130.

11. Kumar S, Kumar S, Gupta B. Urban health: Needs urgent attention. Indian J Public Health [Internet]. 2018 Jul 1 [cited 2021 Aug 23];62(3):214–7. Available from: https://pubmed.ncbi.nlm.nih.gov/30232971/

For peer review only

For peer review only - http://bmjopen.bmj.com/site/about/guidelines.xhtml

12. Bashar MA, Bhattacharya S, Tripathi S, Sharma N, Singh A. Strengthening primary health care through e-referral system. J Family Med Prim Care [Internet]. 2019 [cited 2021 Aug 23];8(4):1511–3. Available from: https://pubmed.ncbi.nlm.nih.gov/31143753/

13. Gidwani S, Jena NN. Perspective article for European Journal of Emergency Medicine: emergency medicine in India. Eur J Emerg Med [Internet]. 2020 [cited 2021 Aug 23];27(5):317–8. Available from: https://pubmed.ncbi.nlm.nih.gov/32852409/

14. McHugh M, Tanabe P, McClelland M, Khare RK. More patients are triaged using the Emergency Severity Index than any other triage acuity system in the United States. Acad Emerg Med [Internet]. 2012 Jan [cited 2021 Aug 23];19(1):106–9. Available from: https://pubmed.ncbi.nlm.nih.gov/22211429/

15. Gerdin LW, Khajanchi M, Kumar V, Roy N, Saha ML, Soni KD, et al. Comparison of emergency department trauma triage performance of clinicians and clinical prediction models: a cohort study in India. BMJ Open [Internet]. 2020 Feb 18 [cited 2021 Aug 23];10(2). Available from: https://pubmed.ncbi.nlm.nih.gov/32075827/

16. Kumar A, Lakshminarayanan D, Joshi N, Vaid S, Bhoi S, Deorari A. Triaging the triage: reducing waiting time to triage in the emergency department at a tertiary care hospital in New Delhi, India. Emerg Med J [Internet]. 2019 Sep 1 [cited 2021 Aug 23];36(9):558–63. Available from: https://pubmed.ncbi.nlm.nih.gov/31366625/

17. Mahajan V, Arora S, Kaur T, Gupta S, Guglani V. Unexpected hospitalisations at a 23-hour observation unit in a paediatric emergency department of northern India. J Clin Diagn Res [Internet]. 2013 Jul 1 [cited 2021 Aug 23];7(7):1418–20. Available from: https://pubmed.ncbi.nlm.nih.gov/23998079/

18. Rominski S, Bell SA, Oduro G, Ampong P, Oteng R, Donkor P. The implementation of the South African Triage Score (SATS) in an urban teaching hospital, Ghana. Afr J Emerg Med [Internet]. 2014 [cited 2021 Aug 23];4(2):71–5. Available from: https://pubmed.ncbi.nlm.nih.gov/28344927/

19. Bruijns SR, Wallis LA, Burch VC. A prospective evaluation of the Cape triage score in the emergency department of an urban public hospital in South Africa. Emerg Med J [Internet]. 2008 Jul [cited 2021 Aug 23];25(7):398–402. Available from: https://pubmed.ncbi.nlm.nih.gov/18573947/

20. World Health Organization. The international statistical classification of diseases and health related problems ICD-10: Tabular list v. 1: Tenth revision. 2nd ed. Genève, Switzerland: World Health Organization. 2005;

21. Mena JH, Sanchez AI, Rubiano AM, Peitzman AB, Sperry JL, Gutierrez MI, et al. Effect of the modified Glasgow Coma Scale score criteria for mild traumatic brain injury on mortality prediction: comparing classic and modified Glasgow Coma Scale score model scores of 13. J Trauma [Internet]. 2011 Nov [cited 2021 Aug 23];71(5):1185–93. Available from: https://pubmed.ncbi.nlm.nih.gov/22071923/

22. Javali RH, Krishnamoorthy, Patil A, Srinivasarangan M, Suraj, Sriharsha. Comparison of Injury Severity Score, New Injury Severity Score, Revised Trauma Score and Trauma and Injury Severity Score for Mortality Prediction in Elderly Trauma Patients. Indian J Crit Care Med [Internet]. 2019 [cited 2021 Aug 23];23(2):73–7. Available from: https://pubmed.ncbi.nlm.nih.gov/31086450/

For peer review only - http://bmjopen.bmj.com/site/about/guidelines.xhtml

23. Champion HR, Sacco WJ, Copes WS, Gann DS, Gennarelli TA, Flanagan ME. A revision of the Trauma Score. J Trauma [Internet]. 1989 [cited 2021 Aug 23];29(5):623–9. Available from: https://pubmed.ncbi.nlm.nih.gov/2657085/

24. Fitschen-Oestern S, Lippross S, Lefering R, Klüter T, Weuster M, Franke GM, et al. Does the time of the day affect multiple trauma care in hospitals? A retrospective analysis of data from the TraumaRegister DGU®. Available from: https://doi.org/10.1186/s12873-021-00525-0

25. R Core Team. R: A Language and Environment for Statistical Computing. Vienna, Austria: R Foundation for Statistical Computing. 2020; Available from: https://www.r-project.org/

26. Ryösä A, Tainio J, Itälä A, Gullichsen E. Clinical Profile, Treatment Details and Survival of Trauma Patients Treated at Intensive Care Unit in a Level II Trauma Center. J Acute Med [Internet]. 2017 [cited 2022 Sep 21];7(1):19–23. Available from: https://doi.org/10.6705/j.jacme.2017.0701.003

27. Sarang B, Bhandarkar P, Raykar N, O'Reilly GM, Soni KD, Wärnberg MG, et al. Associations of On-arrival Vital Signs with 24-hour In-hospital Mortality in Adult Trauma Patients Admitted to Four Public University Hospitals in Urban India: A Prospective Multi-Centre Cohort Study. Injury [Internet]. 2021 May 1 [cited 2021 Aug 23];52(5):1158–63. Available from: https://pubmed.ncbi.nlm.nih.gov/33685640/

Figure 1: Study Flowchart

Figure 2: Percentage distribution of different injury mechanisms among injury types (N = 4135)

Figure 3: Patients' disposition from ED as per retrospective triage using CTS (N=4135)

For peer review only - http://bmjopen.bmj.com/site/about/guidelines.xhtml

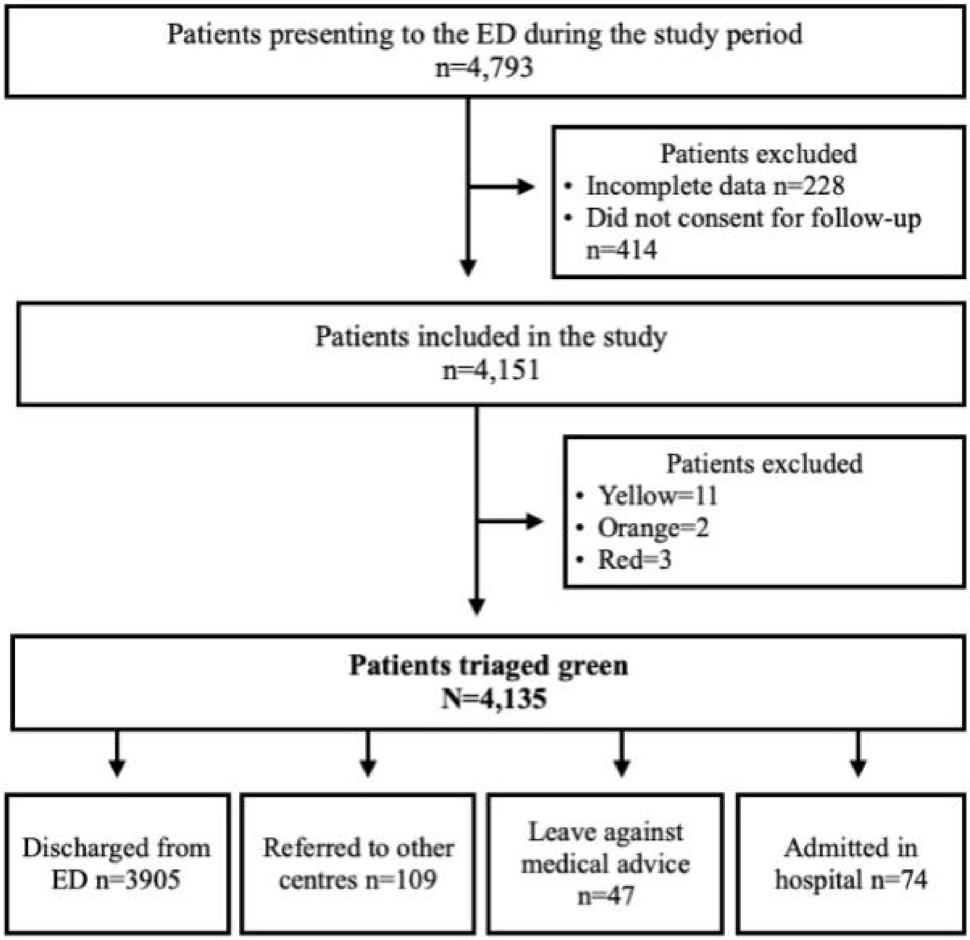

Figure 1: Study Flowchart

52x51mm (300 x 300 DPI)

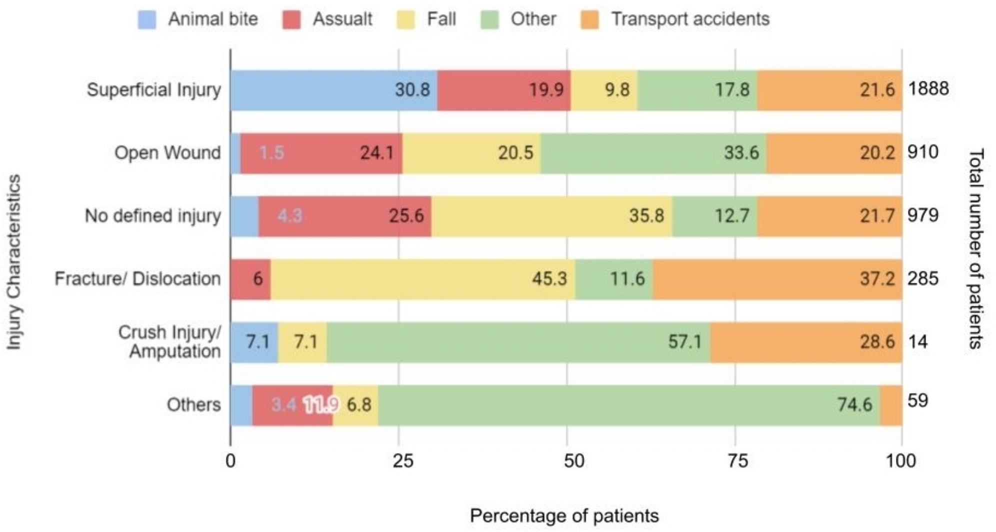

Figure 2: Percentage distribution of different injury mechanisms among injury types (N = 4135)

77x41mm (300 x 300 DPI)

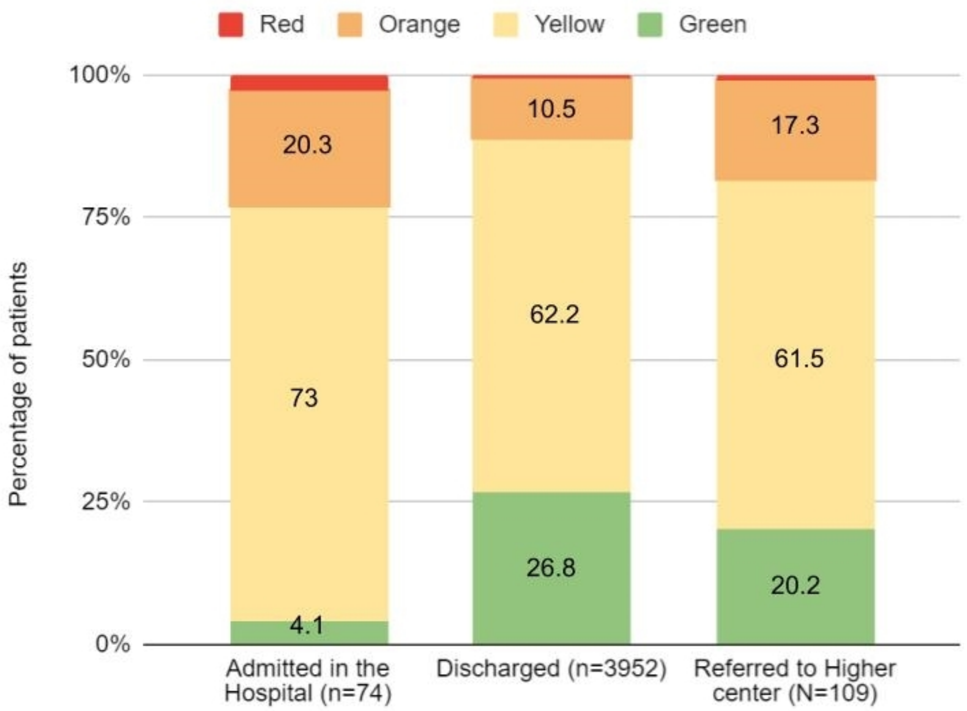

Figure 3: Patients' disposition from ED as per retrospective triage using CTS (N=4135)

61x46mm (300 x 300 DPI)

Table 1: Physiological and injury characteristics of patients triaged green (N=4135)

**Demographics**	
Mechanism of Injury (%)	
Transport accidents	916 (22.2)
Assault	870 (21)
Fall	856 (20.7)
Other	852 (20.6)
Animal bite	641 (15.5)
Transfer status	
Direct	4006 (97)
Transferred	129 (3)
**Vitals**	
AVPU (%)	
Unresponsive	3 (0.1)
Pain	6 (0.1)
Verbal	3 (0.1)
Alert	4123 (99.7)
GCS (mean (SD))	14.98 (0.4)
GCS (%)	
Mild	4125 (99.8)
Moderate	4 (0.1)
Severe	6 (0.1)
Systolic blood pressure (mean (SD))	128.05 (18.9)
Diastolic blood pressure (mean (SD))	84.34 (13.3)
Heart rate (mean (SD))	88.88 (17)
Oxygen saturation (mean (SD))	97.79 (2.2)
Respiratory rate (mean (SD))	22.63 (3.7)
Need for oxygen support (%)	
Not on oxygen support	4135 (100.0)
RTS (mean (SD))	7.99 (0.13)
**Injury characteristics**	
Type of Injury (%)	
Blunt	4075 (98.5)

Penetrating	56 (1.4)
Blunt & penetrating	4 (0.1)
Number of serious injury (%)*	
No serious injury	4112 (99.4)
Single	21 (0.5)
Multiple	2 (0.0)
ISS (%)	
No defined ISS	2048 (49.5)
Mild	2072 (50.1)
Moderate	15 (0.4)
Severe	0 (0.0)
Profound	0 (0.0)

*A serious injury was defined as an injury that warrants hospitalisation.(1)

Table 2A: **The Cape Triage Score depicting the TEWS** (2)

	Adult Triage Score						
	3	2	1	0	1	2	3
Mobility				Walking	With help	Stretcher/ Immobile	
RR		Less than 9		9-14	15-20	21-29	More than 29
HR		Less than 41	41-50	51-100	101-110	111-129	More than 129
SBP	Less than 71	71-80	81-100	101-199		More than 199	
Temp.		Less than 35		35-38.4		38.5 or more	
AVPU				Alert	Reacts to Voice	Reacts to Pain	Unresponsive
Trauma				No	Yes		
**Over 12 years / taller than 150 cm**							

Table 2B: The Cape Triage Score depicting the SATS color code (2)

Colour	Red	Orange	Yellow	Green
TEWS	7 or more	5 - 6	3-4	0-2
Target time to treat	Immediate	Less than 10 min	Less than 60 min	Less than 240 min
Mechanism of injury		High energy transfer		
Presentation		Shortness of breath - acute		All other patients
		Coughing blood		
		Chest pain		
		Haemorrhage uncontrolled	Haemorrhage - controlled	
	Seizure - current	Seizure-post ictal		
		Focal neurology - acute		
		Level of consciousness reduced		
		Psychosis/aggression		
		Threatened limb		
		Dislocation - other joint	Dislocation - finger or toe	
		Fracture - compound	Fracture - closed	
		Burn over 20%	Burn - other	
	Burn-face/ inhalation	Burn - electrical		
		Burn-circumferential		

		Burn-chemical		
		Poisoning/overdose	Abdominal pain	
	Hypoglycemia glucose less than 3	Diabetic - glucose over 11 & ketonuria	Diabetic - glucose over 17 (no ketonuria)	
		Vomiting - fresh blood	Vomiting - persistent	
		Pregnancy and abdominal trauma or pain	Pregnancy and trauma	
			Pregnancy and PV bleed	
Pain		Severe	Moderate	Mild
Senior health care professional's discretion				

References

1. Gardner A, Forson PK, Oduro G, Stewart B, Dike N, Glover P, et al. Diagnostic accuracy of the Kampala Trauma Score using estimated Abbreviated Injury Scale scores and physician opinion. Injury [Internet]. 2017 Jan 1 [cited 2021 Aug 23];48(1):177–83. Available from: https://pubmed.ncbi.nlm.nih.gov/27908493/

2. Rominski S, Bell SA, Oduro G, Ampong P, Oteng R, Donkor P. The implementation of the South African Triage Score (SATS) in an urban teaching hospital, Ghana. African journal of emergency medicine : Revue africaine de la medecine d’urgence [Internet]. 2014 [cited 2021 Aug 23];4(2):71–5. Available from: https://pubmed.ncbi.nlm.nih.gov/28344927/

STROBE 2007 (v4) Statement—Checklist of items that should be included in reports of *cohort studies*

Section/Topic	Item #	Recommendation	Reported on page #
**Title and abstract**	1	(*a*) Indicate the study's design with a commonly used term in the title or the abstract	1
		(*b*) Provide in the abstract an informative and balanced summary of what was done and what was found	3
**Introduction**			
Background/rationale	2	Explain the scientific background and rationale for the investigation being reported	5
Objectives	3	State specific objectives, including any prespecified hypotheses	6
**Methods**			
Study design	4	Present key elements of study design early in the paper	6
Setting	5	Describe the setting, locations, and relevant dates, including periods of recruitment, exposure, follow-up, and data collection	6
Participants	6	(*a*) Give the eligibility criteria, and the sources and methods of selection of participants. Describe methods of follow-up	8
		(*b*) For matched studies, give matching criteria and number of exposed and unexposed	-
Variables	7	Clearly define all outcomes, exposures, predictors, potential confounders, and effect modifiers. Give diagnostic criteria, if applicable	9 - 10
Data sources/ measurement	8*	For each variable of interest, give sources of data and details of methods of assessment (measurement). Describe comparability of assessment methods if there is more than one group	8
Bias	9	Describe any efforts to address potential sources of bias	-
Study size	10	Explain how the study size was arrived at	-
Quantitative variables	11	Explain how quantitative variables were handled in the analyses. If applicable, describe which groupings were chosen and why	12-13
Statistical methods	12	(*a*) Describe all statistical methods, including those used to control for confounding	12-13
		(*b*) Describe any methods used to examine subgroups and interactions	-
		(*c*) Explain how missing data were addressed	12
		(*d*) If applicable, explain how loss to follow-up was addressed	-
		(*e*) Describe any sensitivity analyses	-
**Results**			

For peer review only

Participants	13*	(a) Report numbers of individuals at each stage of study—eg numbers potentially eligible, examined for eligibility, confirmed eligible, included in the study, completing follow-up, and analysed	13
		(b) Give reasons for non-participation at each stage	-
		(c) Consider use of a flow diagram	Figure 1
Descriptive data	14*	(a) Give characteristics of study participants (eg demographic, clinical, social) and information on exposures and potential confounders	13
		(b) Indicate number of participants with missing data for each variable of interest	-
		(c) Summarise follow-up time (eg, average and total amount)	-
Outcome data	15*	Report numbers of outcome events or summary measures over time	15 -16
Main results	16	(*a*) Give unadjusted estimates and, if applicable, confounder-adjusted estimates and their precision (eg, 95% confidence interval). Make clear which confounders were adjusted for and why they were included	-
		(*b*) Report category boundaries when continuous variables were categorized	-
		(*c*) If relevant, consider translating estimates of relative risk into absolute risk for a meaningful time period	-
Other analyses	17	Report other analyses done—eg analyses of subgroups and interactions, and sensitivity analyses	16-17
**Discussion**			
Key results	18	Summarise key results with reference to study objectives	17-20
**Limitations**			
Interpretation	20	Give a cautious overall interpretation of results considering objectives, limitations, multiplicity of analyses, results from similar studies, and other relevant evidence	20
Generalisability	21	Discuss the generalisability (external validity) of the study results	20
**Other information**			
Funding	22	Give the source of funding and the role of the funders for the present study and, if applicable, for the original study on which the present article is based	22

*Give information separately for cases and controls in case-control studies and, if applicable, for exposed and unexposed groups in cohort and cross-sectional studies.

Note: An Explanation and Elaboration article discusses each checklist item and gives methodological background and published examples of transparent reporting. The STROBE checklist is best used in conjunction with this article (freely available on the Web sites of PLoS Medicine at http://www.plosmedicine.org/, Annals of Internal Medicine at http://www.annals.org/, and Epidemiology at http://www.epidem.com/). Information on the STROBE Initiative is available at www.strobe-statement.org.

For peer review only

Profile and Triage Validity of Trauma Patients Triaged Green: A Prospective Cohort Study from a Secondary Care Hospital in India

Journal:	*BMJ Open*
Manuscript ID	bmjopen-2022-065036.R2
Article Type:	Original research
Date Submitted by the Author:	20-Dec-2022
Complete List of Authors:	Anthony, Aroke; Doctors For You<br>Dutta, Rohini; - World Health Organization Collaborating Center for Research in Surgical Care Delivery in Low-and-Middle Income Countries; - Christian Medical College and Hospital,<br>Sarang, Bhakti; World Health Organization Collaborating Centre on Research in Surgical Care Delivery in Low-Middle-Income Countries; Terna Medical College & Hospital, Department of Surgery<br>David, Siddarth; Karolinska Institutet, Department of Global Public Health<br>O'Reilly, Gerard; Monash University, Emergency medicine<br>Raykar, Nakul; Harvard Medical School, Department of Global Health and Social Medicine; Brigham and Women's Hospital, Emergency Surgery<br>Khajanchi, Monty; Seth Gowardhandas Sunderdas Medical College and King Edward Memorial Hospital, Department of Surgery<br>Attergrim, Jonatan; Karolinska Institutet<br>Soni, Kapil; All India Institute of Medical Sciences, Critical Care<br>Sharma, Naveen; All India Institute of Medical Sciences, Surgery<br>Mohan, Monali; WHO Collaboration Centre for Research in Surgical Care Delivery in LMICs, Mumbai, India,<br>Gadgil, Anita; World Health Organization Collaborating Center for Research in Surgical Care Delivery in Low-and-Middle Income Countries<br>Roy, Nobhojit; World Health Organization Collaborating Center for Research in Surgical Care Delivery in Low-and-Middle Income Countries<br>Gerdin Wärnberg, Martin ; Karolinska Institutet, Department of Global Public Health; Karolinska University Hospital
**Primary Subject Heading**:	Emergency medicine
Secondary Subject Heading:	Emergency medicine, Public health
Keywords:	TRAUMA MANAGEMENT, ACCIDENT & EMERGENCY MEDICINE, Trauma management < ORTHOPAEDIC & TRAUMA SURGERY

For peer review only - http://bmjopen.bmj.com/site/about/guidelines.xhtml

For peer review only - http://bmjopen.bmj.com/site/about/guidelines.xhtml

I, the Submitting Author has the right to grant and does grant on behalf of all authors of the Work (as defined in the below author licence), an exclusive licence and/or a non-exclusive licence for contributions from authors who are: i) UK Crown employees; ii) where BMJ has agreed a CC-BY licence shall apply, and/or iii) in accordance with the terms applicable for US Federal Government officers or employees acting as part of their official duties; on a worldwide, perpetual, irrevocable, royalty-free basis to BMJ Publishing Group Ltd ("BMJ") its licensees and where the relevant Journal is co-owned by BMJ to the co-owners of the Journal, to publish the Work in this journal and any other BMJ products and to exploit all rights, as set out in our licence.

The Submitting Author accepts and understands that any supply made under these terms is made by BMJ to the Submitting Author unless you are acting as an employee on behalf of your employer or a postgraduate student of an affiliated institution which is paying any applicable article publishing charge ("APC") for Open Access articles. Where the Submitting Author wishes to make the Work available on an Open Access basis (and intends to pay the relevant APC), the terms of reuse of such Open Access shall be governed by a Creative Commons licence – details of these licences and which Creative Commons licence will apply to this Work are set out in our licence referred to above.

Other than as permitted in any relevant BMJ Author's Self Archiving Policies, I confirm this Work has not been accepted for publication elsewhere, is not being considered for publication elsewhere and does not duplicate material already published. I confirm all authors consent to publication of this Work and authorise the granting of this licence.

For peer review only

Profile and Triage Validity of Trauma Patients Triaged Green: A Prospective Cohort Study from a Secondary Care Hospital in India

Aroke Anna Anthony,[1,2] Rohini Dutta,[2,3] Bhakti Sarang,[2,4] Siddarth David,[5] Gerard O'Reilly,[6,7,8] Nakul Raykar,[9,10,11] Monty Khajanchi,[2,12] Jonatan Attergrim,[5,13] Kapil Dev Soni,[14] Naveen Sharma,[15] Monali Mohan,[2] Anita Gadgil,[2] Nobhojit Roy,[2,5,7] Martin Gerdin Wärnberg[5,13]

[1] Doctors For You, Mumbai, India

[2] Trauma Research Group, World Health Organization Collaborating Centre on Research in Surgical Care Delivery in Low-Middle-Income Countries, Mumbai, India

[3] MBBS, Christian Medical College and Hospital, Ludhiana, India

[4] Department of Surgery, Terna Medical College & Hospital, New Mumbai, India

[5] Department of Global Public Health, Karolinska Institutet, Stockholm, Sweden

[6] Epidemiology & Biostatistics, National Trauma Research Institute, The Alfred, Melbourne, Australia

[7] School of Public Health & Preventive Medicine, Monash University, Melbourne, Australia

[8] Global programs, Emergency & Trauma Centre, The Alfred, Melbourne, Australia

[9] Program in Global Surgery and Social Change, Harvard Medical School, Boston, USA

[10] Trauma, Emergency Surgery & Surgical Critical Care, Brigham and Women's Hospital, Boston, MA, USA

[11] Center for Surgery and Public Health, Brigham and Women's Hospital, Boston, MA, USA

[12] Department of Surgery, Seth.G.S. Medical College & K.E.M. Hospital, Parel, Mumbai, India

[13] Function Perioperative Medicine and Intensive Care, Karolinska University Hospital, Solna, Sweden

[14] Critical & Intensive Care, JPN Apex Trauma Centre, AIIMS, New Delhi, India

[15] Department of Surgery, All India Institute of Medical Sciences, Jodhpur, India

Corresponding Author: Martin Gerdin Wärnberg

Department of Global Public Health, Karolinska Institutet, Stockholm, Sweden; Function Perioperative Medicine and Intensive Care, Karolinska University Hospital, Solna, Sweden.

Address: Department of Global Public Health
Karolinska Institutet
171 77 Stockholm
Email: martin.gerdin@ki.se
Phone Number: +46 708539598

Short title: Profile and Triage Appropriateness of Trauma Patients Triaged Green in a Secondary Care Hospital in India

Word Count (Abstract): 228 words

For peer review only - http://bmjopen.bmj.com/site/about/guidelines.xhtml

Word Count (Manuscript): 2860 words

For peer review only

For peer review only - http://bmjopen.bmj.com/site/about/guidelines.xhtml

ABSTRACT

Objective: To evaluate the profile of non-urgent patients triaged 'green', as part of a triage-trial in the Emergency Department (ED) of a secondary-care hospital in India. The secondary aim was to validate the triage-trial with the South African Triage Score (SATS).

Design: Prospective cohort study

Setting: A secondary care-hospital in Mumbai, India.

Participants: Patients aged 18 years and above with a history of trauma defined as having any of the external causes of morbidity and mortality listed in block V01-Y36, chapter XX of the International Classification of Disease version 10 (ICD-10) codebook, triaged green between July 2016 to November 2019.

Outcome: Outcome measures were mortality within 24-hours, 30-days and mistriage.

Results: We included 4135 trauma patients triaged green. The mean age of patients was 32.8 (±13.1) years, and 77% were males. The median (IQR) length of stay of admitted patients was 3 (13) days. Half the patients had a mild Injury Severity Score (3-8), with the majority of injuries being blunt (98%). Of the patients triaged green by clinicians, three-quarters (74%) were undertriaged on validating with SATS. On telephonic follow-up two patients were reported dead whereas one died while admitted in-hospital.

Conclusion: Our study highlights the need for implementation and evaluation of training in trauma triage systems that use physiological parameters including pulse, systolic blood pressure and glasgow coma scale, for the in-hospital first responders in the EDs.

For peer review only - http://bmjopen.bmj.com/site/about/guidelines.xhtml

Strengths and limitations of this study:

- This is a prospective cohort study, with vital signs recorded by a dedicated research officer, documenting the profile of green triaged patients from a public secondary care hospital in an urban LMIC setting conducted over a period of 3 years.
- Triage validity was assessed using a standardised and validated triage scoring system (SATS), that included both physiological parameters and injury characteristics of the patients.
- The study provides data from a single secondary care hospital in Mumbai. Therefore, the results cannot be generalised to other Indian hospitals due to hospital bias.
- Only the first 10 consecutive patients' data were collected each shift during the study period.
- Data on 30-day mortality was missing for some patients while we have no data on patient morbidity.

For peer review only

INTRODUCTION

Globally, 4.4 million people die from trauma annually, with India contributing to 20% of this burden. [1] Trauma represents the second most common cause of death after age five in India with the majority of deaths attributable to road traffic-related deaths.[2,3] With 50% of deaths due to trauma occurring in the hospitals, there in an urgent need to strengthen in-hospital care for trauma patients.[4]

Trauma care is highly time-sensitive.[5] Hospital triage systems can ensure that critically ill patients are identified and receive care promptly.[6] Several triage scores are used across different countries and hospital settings. [7,8]

India's high population density, poorly developed prehospital care and a lack of appropriate referral systems leads to overcrowding in the emergency departments (EDs). [9–12] Most EDs lack triage protocols and the level of emergency patient care is decided by clinicians who are not trained specifically in trauma care.[13,14] The overcrowding diverts resources from patients requiring immediate care.

In our study, clinicians at a triage-naive ED were introduced to a triage-trial, as part of a multicentre triage project, the Trauma Triage Study in India (TTRIS). TTRIS compared prediction models for triage in adult trauma patients presenting to various emergency departments across India.[15] In TTRIS, the patients were tacitly designated one of the four trauma triage categories by clinicians, based on their understanding of trauma triage; into red, orange, yellow, green, with red and green denoting the most and least urgent patient status respectively.

We aimed to evaluate the profile of the non-urgent patients who were triaged green by clinicians and retrospectively compare the validity of this category using the South African Triage Score (SATS). [16,17]

For peer review only

For peer review only - http://bmjopen.bmj.com/site/about/guidelines.xhtml

METHODOLOGY

Study Design

This single-centre prospective cohort study between July 2016 to November 2019 is part of the TTRIS which compares prediction models for triage in adult trauma patients presenting to various emergency departments across India.

Study Setting

The study site was the ED of Khurshedji Behramji Bhabha Hospital (KBBH), a 436 bedded regional secondary healthcare centre located in Mumbai, India, catering to approximately 350 patients each day in the ED. It is a public hospital with free or nominal fees, providing access to low socio-economic groups and receives patients from across the city. At KBBH, trauma care is imparted as a subspeciality along with medical, surgical, and obstetric care. The hospital has an intensive care facility but there is no neurosurgery department, so patients in need of neurosurgical management are referred to tertiary care centres. Plain radiography and ultrasonography are available round the clock; however computerised tomography (CT) is only available in-house from 7am to 6pm. The patients arriving at the ED are first seen by a casualty medical officer (CMO) largely on a first-come, first-served basis without a formalised system of triaging patients.

Clinician's Tacit Triage (CTT)

As part of data collection of TTRIS, the triage-naive clinicians were only given standard comparable labels to different trauma triage colour categories, without provision to any formal tool or training about the same. The clinicians involved have a minimum of 2 years clinical experience, however, they are neither trained in trauma care as a speciality nor are they necessarily trained in trauma management courses like Advanced Trauma Life Support (ATLS). After their initial on-arrival assessment of each patient, the research officers asked the clinicians to categorise the urgency of patients into the aforementioned colour-coded triage groups,[15] henceforth referred to as the CTT. The CTT was just based on the clinician's experiential and intuitive clinical knowledge. For doing this, the clinicians were

For peer review only - http://bmjopen.bmj.com/site/about/guidelines.xhtml

allowed to use all available information that was extracted by them during initial routine assessment. The CTTs were not used to determine treatment decisions in the ED as there was no formalised tool or protocol in place for assigning the triage and coupling it with patient management. The clinicians were individually informed about the aim and methodology used for the TTRIS study; however, the clinicians were neither involved in the conception nor were they part of the research team analysing the results.

Participants

Inclusion Criteria: We included all the patients aged 18 years and above presenting to the KBBH ED with a history of trauma as their primary complaint and triaged green by clinical triage on initial evaluation irrespective of their injury severity. A history of trauma was defined here as having any of the external causes of morbidity and mortality listed in block V01-Y36, chapter XX of the International Classification of Disease version 10 (ICD-10) online codebook, with some exclusions (see online supplementary material).[18]

Exclusion Criteria: Patients with missing data in one or more variables used for analysis or who did not consent to follow-up were excluded from the analysis.

Source and methods of selection of participants and follow-up

The research officer at KBBH observed morning, evening and night shifts (6-hour observational shifts). These shifts were not aligned with the working hours of the clinical staff to reduce bias and accounting for shift fatigue of the clinicians. Due to the large patient load and time and budgetary constraints of the project, data were collected from the first 10 consecutive patients only, irrespective of their CTT, who presented during each shift. The research officer collected the vital signs but was in no way involved in patient assessment or management.

The research officer performed follow-up at 24-hours, 30-days after arrival at the ED. The follow-up was completed in-person or by telephone, depending on whether the patient was still hospitalised or if the patient had been discharged. The phone numbers of one or more contact persons, mostly relatives, were collected on enrolment and those people were contacted if the participant did not reply to the follow-up telephone

For peer review only - http://bmjopen.bmj.com/site/about/guidelines.xhtml

calls. The outcome was recorded as missing if neither the patient nor the relative were available for follow-up at the specified time-points.

Variables collected for retrospective assessment

To evaluate the profile of patients triaged green we analysed the 24 hours and 30 days mortality, age, sex, mechanism of injury, injury-related details, assigned CTT level, ward or intensive care unit (ICU) admission status, and physiological measures. The physiological measures were systolic blood pressure (SBP), respiratory rate (RR), heart rate (HR), peripheral capillary oxygen saturation (SpO_2), Glasgow Coma Scale (GCS) and Alert Verbal Pain unresponsive scale (AVPU).

GCS was categorised into no or mild traumatic brain injury (TBI) (13-15), moderate TBI (9-12), severe TBI (3-8).[19] Length of stay in the hospital (LoS) was calculated using the data and time of admission in the hospital to the data and time of discharge alive from the hospital, mortality, leave against medical advice (LAMA) or abscond. Injury severity score (ISS) was allocated retrospectively with 'mild' (3-8), 'moderate' (9-15), 'severe' (16-25) and 'profound' (>25) categories. Patients for whom ISS could not be coded, for example when there were no recorded injuries, were assigned 'no defined ISS'.[20] The revised trauma score (RTS) which includes GCS, SBP, and RR and excludes capillary refill and respiratory expansion, which were difficult to assess in the field was computed and categorised as RTS < 4 and RTS > 4.[20,21]

Injuries were recorded and coded using ICD-10 in the TTRIS dataset. Patients were divided into categories with respect to the most critical injury namely, crush injury, injury to internal organs, blood vessel injury, amputation, fracture, dislocation, burn, multiple injury, unspecified injury, open wound, superficial injury.[18] Injury characteristics of patients that presented to the ED with no injuries were categorised as 'no defined injury'. For patients with multiple injuries, the more critical one was considered for categorising patients as per injury. Time of arrival of patients was categorised into four groups, namely, morning (6am-11:59am), afternoon (12pm – 5:59pm), evening (6pm – 11:59pm), and night (12am – 5:59 am).[22]

To determine the validity of CTT, we retrospectively used SATS. SATS has two components, Triage Early Warning Score (TEWS) which uses the physiological parameters and the SATS clinical discriminators

For peer review only - http://bmjopen.bmj.com/site/about/guidelines.xhtml

(SATScd) that use pathology of the patient to triage.[16] Retrospectively calculated variables and triage categories are henceforth labelled with a prefix r, for example, rSATS. First the rTEWS was calculated and then matched for rSATScd. If a clinical discriminator, such as fracture or dislocation, was present the rSATS was updated to match the triage level assigned to each SATScd (See online supplementary material), to be classified into rRed, rOrange, rYellow and rGreen.

Bias

There were three layers of quality control. First, data was entered using a dedicated electronic data collection instrument with extensive logical checks and prompts for unlikely but possible values. Second, the collected data were reviewed on a weekly basis and discussed during weekly online conferences with all research officers and the project leads throughout the duration of the data collection period. Third, on-site quality control sessions were conducted every 3-4 months. During these sessions, a second research officer collected data alongside the research officer who worked at the ED. The quality-controlled data was then compared with the standard data.

Patient and Public Involvement

No patients were involved in the design of the study.

Statistical Methods

Data was analysed using R version 4.04 statistical software.[23] Complete case analysis was performed to only include patients with complete data. We describe the sample using frequencies and percentages for categorical variables, and mean and standard deviation (SD) for normally distributed continuous variables and median and inter quartile range (IQR) for non-normally distributed continuous variables. The number of patients triaged green by the SATS was divided by the number of patients triaged green as per clinician triage (4135), the resultant proportion minus one was considered as the proportion of patients mis-triaged.

Ethical clearance

Ethics committee approval for TTRIS was obtained from the ethics and scientific committee of KBBH (KBBH, HO/4982/KBB,12/08/2016).

For peer review only - http://bmjopen.bmj.com/site/about/guidelines.xhtml

RESULTS

In the study, 4151 patients were included of which 4135 (99.6%) patients were triaged green by CTT **(Figure 1)**.

Profile of patients triaged green

The mean age of patients was 32.8±13.1 years with 3172 (77%) males. Notably, of all patients triaged green, 10/4135 (0.24%) patients presented with moderate to severe GCS and 0.3% of patients did not have an AVPU of alert. Majority of patients (97%) triaged green presented to the study-site directly without a primary care hospital referral.

Of the total patients triaged green by CTT, 46% of patients had only superficial injuries of which majority (30.8%) were due to animal bites. Further, 24% had no external injuries on examination. Among those referred to other centres, the most common types of injury identified were superficial injuries (34) followed by open wounds (27) and patients with no documented injury (19). The reasons for referral to other centres were not documented. As per ISS, 50.2% of patients had 'mild' and 0.4% had 'moderate' score and the remaining 49.5% patients had 'no defined ISS'. **Figure 2** shows the different injury types as per mechanism of injury in the study population. Amongst those that had a transport accident, 881/916 (96.17%) were patients who had a road traffic injury.

The ED disposition of all the patients is shown in **Figure 1**. The median (IQR) LoS of those admitted to the hospital was 3 (13) days and seven patients required admission in the ICU. Most admitted patients 62/74 (83.8%) were successfully discharged from the hospital while three were transferred to other centres for further management. Further, there were eight patients that took LAMA and one who died during their hospital stay.

Follow up at 30 days was successful for 3832/4135 (92.7%) of patients. Three patients died during the first 30 days. Of these patients, two had a GCS of <8 on initial evaluation. The rSATS triage of these patients were rYellow and rOrange.

For peer review only - http://bmjopen.bmj.com/site/about/guidelines.xhtml

Evaluation of triage validity through rSATS

We found that of the total number of patients that were triaged green by CTT (N=4135), 24 patients were triaged rRed, 448 patients were triaged rOrange and 2579 patients were triaged rYellow as per rSATS indicating that 73.8% patients were undertriaged by CTT. Proportions of undertriage were higher during the night and afternoon (**Table 1A**). Of these, most patients (97%) were found to have been undertriaged after assessing their physiological parameters from rTEWS while others due to the rSATScd as seen in **Table 1B**. In **Figure 3** the disposition of these patients from the ED as per their rSATS is depicted. Notably, of the total three documented deaths, one occurred in a patient who was admitted in the hospital and triaged rOrange, and one in a patient transferred to a different centre triaged rYellow. **Table 1C** shows the rSATS of patients with fractures, dislocation and amputations.

Table 1 A: Patients undertriaged as per the time of the day [24]

	Morning	Afternoon	Evening	Night	p
n	756	1576	1155	648	
**Undertriage**	511 (67.6)	1185 (75.2)	858 (74.3)	497 (76.7)	<0.001

Table 1 B: Patients undertriaged as per rSATS (N=4135)

rSATS	rGreen	rYellow	rOrange	rRed
n	1084	2579	448	24
**rTEWS**	1084 (100)	2513 (97.4)	433 (96.7)	19 (79.2)
**rSATScd**	0	66 (2.6)	15 (3.3)	5 (20.8)

Table 1 C: Injury characteristics as per the rSATS Triage category

	rSATS	rGreen	rYellow	rOrange	rRed	p
n		1084	2579	448	24	
	Amputation/ Crush Injury	0 (0.0)	13 (0.5)	1 (0.2)	0 (0.0)	<0.001

For peer review only - http://bmjopen.bmj.com/site/about/guidelines.xhtml

	Fracture/Dislocation	0 (0.0)	243 (9.4)	41 (9.2)	1 (4.2)
**Injury Type**	Others	9 (0.8)	36 (1.4)	9 (2.0)	5 (20.8)
	Open wound	204 (18.8)	575 (22.3)	123 (27.5)	8 (33.3)
	Superficial Injury	635 (58.6)	1074 (41.6)	174 (38.8)	5 (20.8)
	No defined Injury	236 (21.8)	638 (24.7)	100 (22.3)	5 (20.8)

DISCUSSION

Main Findings

Blunt trauma was seen as the most common type of injury.[24] Transport accidents was the predominant mechanism of injury and 77% patients were males. Most patients had mild ISS (50.2%) and only about 0.4% patients had moderate ISS with no patients in the severe and profound ISS category. Most patients presented with seemingly superficial injuries. The presentation of 15.5% of patients with animal bites was unique to our setting. These patients mainly presented for vaccinations following animal bites more frequently than for the treatment of bite injuries.

This study shows that approximately three quarters (74%) of patients triaged green by CTT were undertriaged when compared to rSATS. Out of 4135 patients triaged green by CTT only 1084/4135 (26.2%) were triaged green according to the rSATS. Most of these patients (97%) were coming in as direct arrival to this secondary-care hospital. According to CTT, patients were triaged green even with GCS moderate to severe (10/4135, 0.24%) and 0.3% of patients did not have an AVPU of alert and were still triaged green.

In addition to the high proportion of undertriage ascertained by rTEWS, of those admitted, seven required admissions to the ICU indicating they may have required urgent management for their condition. On a closer look at the physiological parameters of the three patients found dead on 30-day follow-up, it was seen that two of them were under triaged on initial evaluation as they had a GCS < 8.

Interpretation and Clinical relevance

For peer review only - http://bmjopen.bmj.com/site/about/guidelines.xhtml

Among the green triaged patients, there were patients whose physiological parameters indicated that they required urgent attention although the proportion of these patients is relatively low compared to our sample size. These findings emphasise the need for an ED triage and an effective referral system based on on-scene triage. Additionally, they highlight the efficacy of physiological scores such as TEWS, a component of SATS, in triaging patients accurately and the need to include GCS assessment for all patients presenting to the ED. Reasons for these patients being undertriaged must be explored extensively to enhance healthcare delivery in the EDs. Although the reasons for this undertriage are multifactorial, in this case, the lack of appropriate training or standard, uniform protocol for patient management in the ED to quickly identify these patients among those that have normal physiological parameters is most evident. The other factor, overcrowding of the ED with limitation of resources, may also lead to inadequate trauma care.[25]

Strengths

This is a prospective cohort study, with vital signs recorded by a dedicated research officer, documenting the profile of green triaged patients from a public secondary care hospital in an urban LMIC setting conducted over a period of 3 years. Triage validity was assessed using a standardised and validated triage scoring system (SATS), that included both physiological parameters and injury characteristics of the patients.

Limitations

Firstly, the study provides data from a single secondary care centre, results of which may not be generalisable to other secondary care hospitals or other Indian healthcare settings. Secondly, to ensure feasibility, data of only 10 consecutive patients were collected in each shift. Thirdly, we did not have data on the number of clinicians that participated in the triaging process or how they acquired knowledge and skills to triage patients. Lastly, 30-day mortality was missing for some patients while we have none on morbidity. This is a limiting factor towards assessing the morbidity gains.

CONCLUSION

For peer review only - http://bmjopen.bmj.com/site/about/guidelines.xhtml

Three-fourths (74%) of the patients triaged green by clinicians in a secondary care hospital in Mumbai were undertriaged when analysed using rSATS. This highlights the need for implementation and evaluation of trauma triage training, involving systems that rely on presenting physiological parameters, for clinicians, nurses and other paramedical staff in the EDs. Additionally, direct admissions of the non-urgent patients to this secondary-care hospital warrants strengthening the referral systems to avoid overcrowding of the Indian EDs.

LIST OF ABBREVIATIONS

Emergency department (ED), Trauma Triage Study in India (TTRIS), South African Triage Scale (SATS), Triage Early Warning Score (TEWS), Low- and Middle-Income Country (LMIC), Khurshedji Behramji Bhabha Hospital (KBBH), Computerized Tomography (CT), Casualty Medical Officer (CMO), Clinician's Tacit Triage (CTT) , International Classification of Disease version 10 (ICD-10), Intensive Care Unit (ICU), Systolic blood pressure (SBP), Respiratory rate (RR), Heart rate (HR), Peripheral capillary oxygen saturation (SpO2), Glasgow Coma Scale (GCS), Alert Verbal Pain unresponsive scaled (AVPU), Traumatic Brain Injury (TBI), Length of stay in the hospital (LoS), Leave against medical advice (LAMA), Injury Severity Score (ISS), Revised Trauma Score (RTS), Retrospective SATS (rSATS), Retrospective TEWS (rTEWS), Retrospective SATS clinical discriminators (rSATScd), Retrospective Green (rGreen), Retrospective Yellow (rYellow), Retrospective Orange (rOrange), Retrospective Red (rRed), Standard deviation (SD), Inter quartile range (IQR)

DECLARATIONS

Ethical clearance

Ethics committee approval for TTRIS was obtained from the ethics and scientific committee of KBBH (KBBH, HO/4982/KBB,12/08/2016). Informed consent for follow-up was taken from patients at the time

For peer review only

of discharge from the hospital. In case the patient was unconscious, consent was obtained from a family member or the patient's legally acceptable representative.

Consent for publication

Not Applicable

Availability of data and materials

The data are available to whoever wants them by emailing the corresponding author. They can write their aims and objectives, and then, the authors can decide if that study can be done without duplication of the work.

Competing Interests

There is no conflict of interest to disclose from any of the authors

Funding

The data collection for TTRIS was funded by grants from the Swedish National Board of Health and Welfare, grant numbers 22464/2017 and 23745/2016.

Author Contribution

Authors AA, RD, BS, SD and MGW have conceptualized the study. AA and RD analysed the data and AA, RD, BS were involved in the interpretation of the data and manuscript writing. GR, NR, MK, JA, KDS, NS, MM, AG, NR, and MGW contributed to the study design and critical revisions to the manuscript. All authors have contributed to drafting the article and revising it. They also approved the final version of the manuscript. All authors agree to be responsible for all aspects of the work.

Acknowledgments

We thank the Thursday Truth Seekers (TTS) and the Trauma Triage Study in India (TTRIS), India, research team for their support.

REFERENCES

1. GBD 2017 Causes of Death Collaborators. Global, regional, and national age-sex-specific mortality for 282 causes of death in 195 countries and territories, 1980-2017: a systematic analysis for the Global

For peer review only - http://bmjopen.bmj.com/site/about/guidelines.xhtml

Burden of Disease Study 2017. Lancet [Internet]. 2018 Nov 10 [cited 2021 Aug 23];392(10159):1736–88. Available from: https://pubmed.ncbi.nlm.nih.gov/30496103/

2. World Health Organization. WHO country cooperation strategy at a glance : India. 2018. Available from: https://apps.who.int/iris/handle/10665/136895

3. Government of India. Ministry of Road Transport & Highways Transport Research Wing. Road Accidents in India 2019. 2019; Available from: https://morth.nic.in/sites/default/files/RA_Uploading.pdf

4. G G. Injuries in India: A national perspective. Burden of Disease in India Equitable Development - Healthy Future New Delhi: National Commission on Macroeconomics and Health, Ministry of Health & Family Welfare, Government of India. 2005;325–347. Available from: http://www.drbrambedkarcollege.ac.in/sites/default/files/Burden of disease in India.pdf#page=343

5. Fitzgerald M, Cameron P, Mackenzie C, Farrow N, Scicluna P, Gocentas R, et al. Trauma resuscitation errors and computer-assisted decision support. Arch Surg [Internet]. 2011 Feb [cited 2021 Aug 23];146(2):218–25. Available from: https://pubmed.ncbi.nlm.nih.gov/21339436/

6. Cameron P, Gabbe B, Smith K, Mitra B. Triaging the right patient to the right place in the shortest time. Br J Anaesth [Internet]. 2014 [cited 2021 Aug 23];113(2):226–33. Available from: https://pubmed.ncbi.nlm.nih.gov/24961786/

7. Twomey M, Wallis LA, Thompson M lou, Myers JE. The South African Triage Scale (adult version) provides reliable acuity ratings. Int Emerg Nurs [Internet]. 2012 Jul [cited 2021 Aug 23];20(3):142–50. Available from: https://pubmed.ncbi.nlm.nih.gov/22726946/

8. Ebrahimi M, Heydari A, Mazlom R, Mirhaghi A. The reliability of the Australasian Triage Scale: a meta-analysis. World J Emerg Med [Internet]. 2015 [cited 2021 Aug 23];6(2):94. Available from: https://pubmed.ncbi.nlm.nih.gov/26056538/

9. Roy N, Murlidhar V, Chowdhury R, Patil SB, Supe PA, Vaishnav PD, et al. Where there are no emergency medical services-prehospital care for the injured in Mumbai, India. Prehosp Disaster Med [Internet]. 2010 [cited 2021 Aug 23];25(2):145–51. Available from: https://pubmed.ncbi.nlm.nih.gov/20467994/

10. Woolard RH, Borron SW MJM. Emergency Department Design. In: Ciottone's Disaster Medicine. Elsevier. 2016;125–130.

11. Kumar S, Kumar S, Gupta B. Urban health: Needs urgent attention. Indian J Public Health [Internet]. 2018 Jul 1 [cited 2021 Aug 23];62(3):214–7. Available from: https://pubmed.ncbi.nlm.nih.gov/30232971/

12. Bashar MA, Bhattacharya S, Tripathi S, Sharma N, Singh A. Strengthening primary health care through e-referral system. J Family Med Prim Care [Internet]. 2019 [cited 2021 Aug 23];8(4):1511–3. Available from: https://pubmed.ncbi.nlm.nih.gov/31143753/

13. Gidwani S, Jena NN. Perspective article for European Journal of Emergency Medicine: emergency medicine in India. Eur J Emerg Med [Internet]. 2020 [cited 2021 Aug 23];27(5):317–8. Available from: https://pubmed.ncbi.nlm.nih.gov/32852409/

14. McHugh M, Tanabe P, McClelland M, Khare RK. More patients are triaged using the Emergency Severity Index than any other triage acuity system in the United States. Acad Emerg Med [Internet]. 2012 Jan [cited 2021 Aug 23];19(1):106–9. Available from: https://pubmed.ncbi.nlm.nih.gov/22211429/

For peer review only

15. Gerdin LW, Khajanchi M, Kumar V, Roy N, Saha ML, Soni KD, et al. Comparison of emergency department trauma triage performance of clinicians and clinical prediction models: a cohort study in India. BMJ Open [Internet]. 2020 Feb 18 [cited 2021 Aug 23];10(2). Available from: https://pubmed.ncbi.nlm.nih.gov/32075827/

16. Rominski S, Bell SA, Oduro G, Ampong P, Oteng R, Donkor P. The implementation of the South African Triage Score (SATS) in an urban teaching hospital, Ghana. Afr J Emerg Med [Internet]. 2014 [cited 2021 Aug 23];4(2):71–5. Available from: https://pubmed.ncbi.nlm.nih.gov/28344927/

17. Bruijns SR, Wallis LA, Burch VC. A prospective evaluation of the Cape triage score in the emergency department of an urban public hospital in South Africa. Emerg Med J [Internet]. 2008 Jul [cited 2021 Aug 23];25(7):398–402. Available from: https://pubmed.ncbi.nlm.nih.gov/18573947/

18. World Health Organization. The international statistical classification of diseases and health related problems ICD-10: Tabular list v. 1: Tenth revision. 2nd ed. Genève, Switzerland: World Health Organization. 2005;

19. Mena JH, Sanchez AI, Rubiano AM, Peitzman AB, Sperry JL, Gutierrez MI, et al. Effect of the modified Glasgow Coma Scale score criteria for mild traumatic brain injury on mortality prediction: comparing classic and modified Glasgow Coma Scale score model scores of 13. J Trauma [Internet]. 2011 Nov [cited 2021 Aug 23];71(5):1185–93. Available from: https://pubmed.ncbi.nlm.nih.gov/22071923/

20. Javali RH, Krishnamoorthy, Patil A, Srinivasarangan M, Suraj, Sriharsha. Comparison of Injury Severity Score, New Injury Severity Score, Revised Trauma Score and Trauma and Injury Severity Score for Mortality Prediction in Elderly Trauma Patients. Indian J Crit Care Med [Internet]. 2019 [cited 2021 Aug 23];23(2):73–7. Available from: https://pubmed.ncbi.nlm.nih.gov/31086450/

21. Champion HR, Sacco WJ, Copes WS, Gann DS, Gennarelli TA, Flanagan ME. A revision of the Trauma Score. J Trauma [Internet]. 1989 [cited 2021 Aug 23];29(5):623–9. Available from: https://pubmed.ncbi.nlm.nih.gov/2657085/

22. Fitschen-Oestern S, Lippross S, Lefering R, Klüter T, Weuster M, Franke GM, et al. Does the time of the day affect multiple trauma care in hospitals? A retrospective analysis of data from the TraumaRegister DGU®. Available from: https://doi.org/10.1186/s12873-021-00525-0

23. R Core Team. R: A Language and Environment for Statistical Computing. Vienna, Austria: R Foundation for Statistical Computing. 2020; Available from: https://www.r-project.org/

24. Ryösä A, Tainio J, Itälä A, Gullichsen E. Clinical Profile, Treatment Details and Survival of Trauma Patients Treated at Intensive Care Unit in a Level II Trauma Center. J Acute Med [Internet]. 2017 [cited 2022 Sep 21];7(1):19–23. Available from: https://doi.org/10.6705/j.jacme.2017.0701.003

25. Sarang B, Bhandarkar P, Raykar N, O'Reilly GM, Soni KD, Wärnberg MG, et al. Associations of On-arrival Vital Signs with 24-hour In-hospital Mortality in Adult Trauma Patients Admitted to Four Public University Hospitals in Urban India: A Prospective Multi-Centre Cohort Study. Injury [Internet]. 2021 May 1 [cited 2021 Aug 23];52(5):1158–63. Available from: https://pubmed.ncbi.nlm.nih.gov/33685640/

Figure 1: Study Flowchart

Figure 2: Percentage distribution of different injury mechanisms among injury types (N = 4135)

For peer review only - http://bmjopen.bmj.com/site/about/guidelines.xhtml

Figure 3: Patients' disposition from ED as per retrospective triage using CTS (N=4135)

For peer review only

For peer review only - http://bmjopen.bmj.com/site/about/guidelines.xhtml

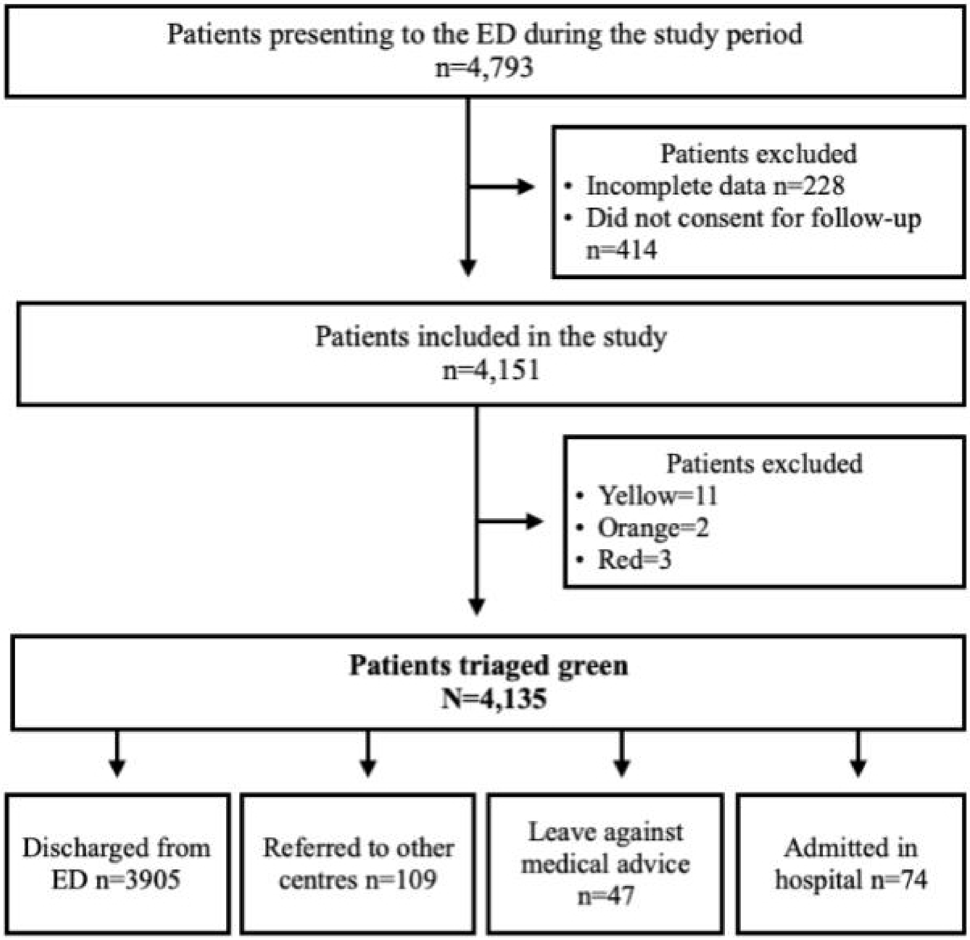

Figure 1: Study Flowchart

52x51mm (300 x 300 DPI)

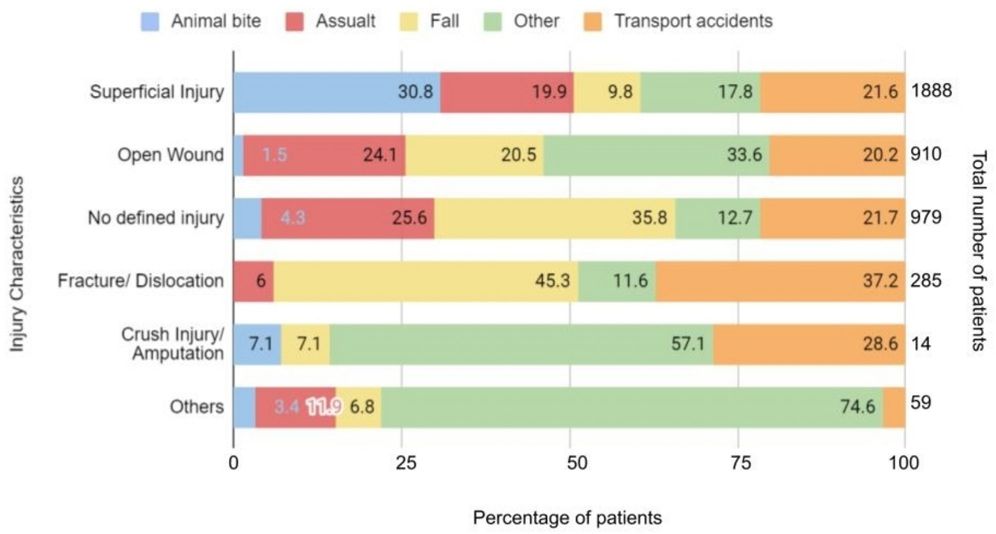

Figure 2: Percentage distribution of different injury mechanisms among injury types (N = 4135)

77x41mm (300 x 300 DPI)

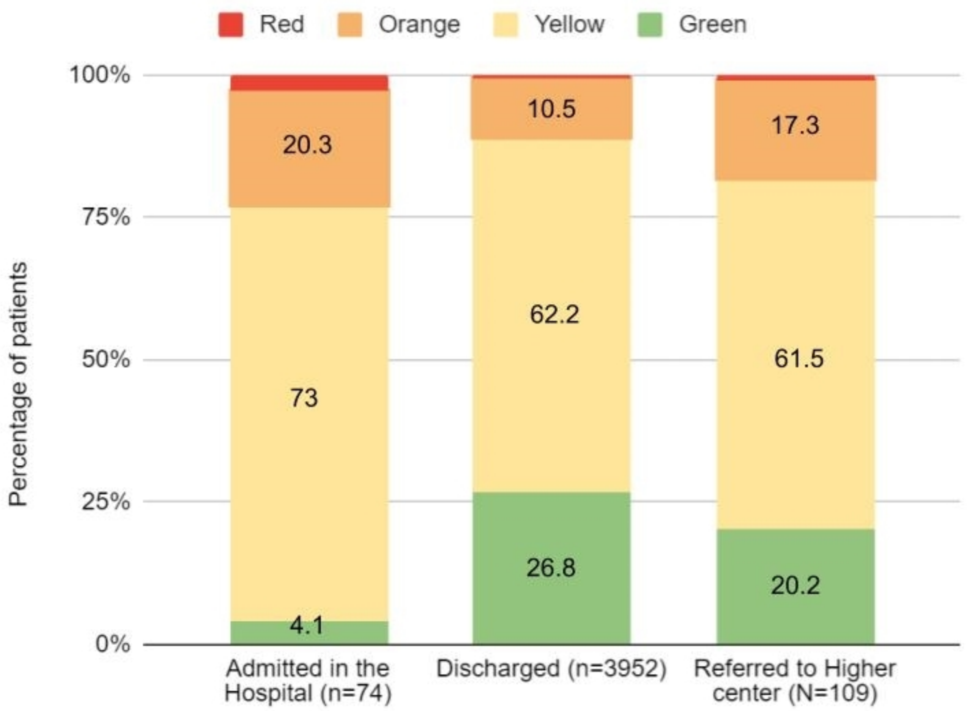

Figure 3: Patients' disposition from ED as per retrospective triage using CTS (N=4135)

61x46mm (300 x 300 DPI)

Table 1: Physiological and injury characteristics of patients triaged green (N=4135)

**Demographics**	
Mechanism of Injury (%)	
Transport accidents	916 (22.2)
Assault	870 (21)
Fall	856 (20.7)
Other	852 (20.6)
Animal bite	641 (15.5)
Transfer status	
Direct	4006 (97)
Transferred	129 (3)
**Vitals**	
AVPU (%)	
Unresponsive	3 (0.1)
Pain	6 (0.1)
Verbal	3 (0.1)
Alert	4123 (99.7)
GCS (mean (SD))	14.98 (0.4)
GCS (%)	
Mild	4125 (99.8)
Moderate	4 (0.1)
Severe	6 (0.1)
Systolic blood pressure (mean (SD))	128.05 (18.9)
Diastolic blood pressure (mean (SD))	84.34 (13.3)
Heart rate (mean (SD))	88.88 (17)
Oxygen saturation (mean (SD))	97.79 (2.2)
Respiratory rate (mean (SD))	22.63 (3.7)
Need for oxygen support (%)	
Not on oxygen support	4135 (100.0)
RTS (mean (SD))	7.99 (0.13)
**Injury characteristics**	
Type of Injury (%)	
Blunt	4075 (98.5)

Penetrating	56 (1.4)
Blunt & penetrating	4 (0.1)
Number of serious injury (%)*	
No serious injury	4112 (99.4)
Single	21 (0.5)
Multiple	2 (0.0)
ISS (%)	
No defined ISS	2048 (49.5)
Mild	2072 (50.1)
Moderate	15 (0.4)
Severe	0 (0.0)
Profound	0 (0.0)

*A serious injury was defined as an injury that warrants hospitalisation.(1)

Table 2A: **The Cape Triage Score depicting the TEWS** (2)

	Adult Triage Score						
	3	2	1	0	1	2	3
Mobility				Walking	With help	Stretcher/ Immobile	
RR		Less than 9		9-14	15-20	21-29	More than 29
HR		Less than 41	41-50	51-100	101-110	111-129	More than 129
SBP	Less than 71	71-80	81-100	101-199		More than 199	
Temp.		Less than 35		35-38.4		38.5 or more	
AVPU				Alert	Reacts to Voice	Reacts to Pain	Unresponsive
Trauma				No	Yes		
**Over 12 years / taller than 150 cm**							

Table 2B: The Cape Triage Score depicting the SATS color code (2)

Colour	Red	Orange	Yellow	Green
TEWS	7 or more	5 - 6	3-4	0-2
Target time to treat	Immediate	Less than 10 min	Less than 60 min	Less than 240 min
Mechanism of injury		High energy transfer		
Presentation		Shortness of breath - acute		All other patients
		Coughing blood		
		Chest pain		
		Haemorrhage uncontrolled	Haemorrhage - controlled	
	Seizure - current	Seizure-post ictal		
		Focal neurology - acute		
		Level of consciousness reduced		
		Psychosis/aggression		
		Threatened limb		
		Dislocation - other joint	Dislocation - finger or toe	
		Fracture - compound	Fracture - closed	
		Burn over 20%	Burn - other	
	Burn-face/ inhalation	Burn - electrical		
		Burn-circumferential		

		Burn-chemical		
		Poisoning/overdose	Abdominal pain	
	Hypoglycemia glucose less than 3	Diabetic - glucose over 11 & ketonuria	Diabetic - glucose over 17 (no ketonuria)	
		Vomiting - fresh blood	Vomiting - persistent	
		Pregnancy and abdominal trauma or pain	Pregnancy and trauma	
			Pregnancy and PV bleed	
Pain		Severe	Moderate	Mild
Senior health care professional's discretion				

References

1. Gardner A, Forson PK, Oduro G, Stewart B, Dike N, Glover P, et al. Diagnostic accuracy of the Kampala Trauma Score using estimated Abbreviated Injury Scale scores and physician opinion. Injury [Internet]. 2017 Jan 1 [cited 2021 Aug 23];48(1):177–83. Available from: https://pubmed.ncbi.nlm.nih.gov/27908493/

2. Rominski S, Bell SA, Oduro G, Ampong P, Oteng R, Donkor P. The implementation of the South African Triage Score (SATS) in an urban teaching hospital, Ghana. African journal of emergency medicine : Revue africaine de la medecine d'urgence [Internet]. 2014 [cited 2021 Aug 23];4(2):71–5. Available from: https://pubmed.ncbi.nlm.nih.gov/28344927/

STROBE Statement—Checklist of items that should be included in reports of ***cohort studies***

	Item No	Recommendation	Page No
**Title and abstract**	1	(*a*) Indicate the study's design with a commonly used term in the title or the abstract	1
		(*b*) Provide in the abstract an informative and balanced summary of what was done and what was found	3
**Introduction**			
Background/rationale	2	Explain the scientific background and rationale for the investigation being reported	5
Objectives	3	State specific objectives, including any prespecified hypotheses	5
**Methods**			
Study design	4	Present key elements of study design early in the paper	6
Setting	5	Describe the setting, locations, and relevant dates, including periods of recruitment, exposure, follow-up, and data collection	6
Participants	6	(*a*) Give the eligibility criteria, and the sources and methods of selection of participants. Describe methods of follow-up<br>(*b*) For matched studies, give matching criteria and number of exposed and unexposed	7
Variables	7	Clearly define all outcomes, exposures, predictors, potential confounders, and effect modifiers. Give diagnostic criteria, if applicable	8
Data sources/ measurement	8*	For each variable of interest, give sources of data and details of methods of assessment (measurement). Describe comparability of assessment methods if there is more than one group	7
Bias	9	Describe any efforts to address potential sources of bias	9
Study size	10	Explain how the study size was arrived at	-
Quantitative variables	11	Explain how quantitative variables were handled in the analyses. If applicable, describe which groupings were chosen and why	9
Statistical methods	12	(*a*) Describe all statistical methods, including those used to control for confounding<br>(*b*) Describe any methods used to examine subgroups and interactions<br>(*c*) Explain how missing data were addressed<br>(*d*) If applicable, explain how loss to follow-up was addressed<br>(*e*) Describe any sensitivity analyses	9
**Results**			
Participants	13*	(a) Report numbers of individuals at each stage of study—eg numbers potentially eligible, examined for eligibility, confirmed eligible, included in the study, completing follow-up, and analysed<br>(b) Give reasons for non-participation at each stage<br>(c) Consider use of a flow diagram	10<br><br><br>Figure 1
Descriptive data	14*	(a) Give characteristics of study participants (eg demographic, clinical, social) and information on exposures and potential confounders<br>(b) Indicate number of participants with missing data for each variable of interest<br>(c) Summarise follow-up time (eg, average and total amount)	10
Outcome data	15*	Report numbers of outcome events or summary measures over time	10-11

For peer review only

Main results	16	(*a*) Give unadjusted estimates and, if applicable, confounder-adjusted estimates and their precision (eg, 95% confidence interval). Make clear which confounders were adjusted for and why they were included<br>(*b*) Report category boundaries when continuous variables were categorized<br>(*c*) If relevant, consider translating estimates of relative risk into absolute risk for a meaningful time period	10-11
Other analyses	17	Report other analyses done—eg analyses of subgroups and interactions, and sensitivity analyses	11
**Discussion**			
Key results	18	Summarise key results with reference to study objectives	12
Limitations	19	Discuss limitations of the study, taking into account sources of potential bias or imprecision. Discuss both direction and magnitude of any potential bias	13
Interpretation	20	Give a cautious overall interpretation of results considering objectives, limitations, multiplicity of analyses, results from similar studies, and other relevant evidence	12-13
Generalisability	21	Discuss the generalisability (external validity) of the study results	13
**Other information**			
Funding	22	Give the source of funding and the role of the funders for the present study and, if applicable, for the original study on which the present article is based	15

*Give information separately for exposed and unexposed groups.

Note: An Explanation and Elaboration article discusses each checklist item and gives methodological background and published examples of transparent reporting. The STROBE checklist is best used in conjunction with this article (freely available on the Web sites of PLoS Medicine at http://www.plosmedicine.org/, Annals of Internal Medicine at http://www.annals.org/, and Epidemiology at http://www.epidem.com/). Information on the STROBE Initiative is available at http://www.strobe-statement.org.

For peer review only

For peer review only - http://bmjopen.bmj.com/site/about/guidelines.xhtml
